# Rapid neutrophil mobilization by VCAM-1+ endothelial cell-derived extracellular vesicles

**DOI:** 10.1093/cvr/cvac012

**Published:** 2022-02-04

**Authors:** Naveed Akbar, Adam T Braithwaite, Emma M Corr, Graeme J Koelwyn, Coen van Solingen, Clément Cochain, Antoine-Emmanuel Saliba, Alastair Corbin, Daniela Pezzolla, Malene Møller Jørgensen, Rikke Bæk, Laurienne Edgar, Carla De Villiers, Mala Gunadasa-Rohling, Abhirup Banerjee, Daan Paget, Charlotte Lee, Eleanor Hogg, Adam Costin, Raman Dhaliwal, Errin Johnson, Thomas Krausgruber, Joey Riepsaame, Genevieve E Melling, Mayooran Shanmuganathan, Oxford Acute Myocardial Infarction Study (OxAMI), Adrian Banning, Raj Kharbanda, Neil Ruparelia, Mohammad Alkhalil, GianLiugi De Maria, Lisa Gaughran, Erica Dall’Armellina, Vanessa Ferreira, Alessandra Borlotti, Yujun Ng, Christoph Bock, David R F Carter, Keith M Channon, Paul R Riley, Irina A Udalova, Kathryn J Moore, Daniel C Anthony, Robin P Choudhury

**Affiliations:** Division of Cardiovascular Medicine, Radcliffe Department of Medicine University of Oxford Level 6, West Wing John Radcliffe Hospital Headington Oxford OX3 9DU, UK; Division of Cardiovascular Medicine, Radcliffe Department of Medicine University of Oxford Level 6, West Wing John Radcliffe Hospital Headington Oxford OX3 9DU, UK; NYU Cardiovascular Research Center, Department of Medicine, Division of Cardiology, School of Medicine, New York University School of Medicine, 435 E 30th St. New York, NY 10016, USA; NYU Cardiovascular Research Center, Department of Medicine, Division of Cardiology, School of Medicine, New York University School of Medicine, 435 E 30th St. New York, NY 10016, USA; NYU Cardiovascular Research Center, Department of Medicine, Division of Cardiology, School of Medicine, New York University School of Medicine, 435 E 30th St. New York, NY 10016, USA; Comprehensive Heart Failure Center, University Hospital Wurzburg, Anstalt des öffentlichen Rechts Josef-Schneider-Straße 2 97080 Würzburg, Germany; Helmholtz Institute for RNA-based Infection Research (HIRI), Helmholtz-Center for Infection Research (HZI), Inhoffenstraße 7 38124 Braunschweig, Würzburg, Germany; Kennedy Institute of Rheumatology, University of Oxford, Roosevelt Dr, Headington, Oxford OX3 7FY, UK; Division of Cardiovascular Medicine, Radcliffe Department of Medicine University of Oxford Level 6, West Wing John Radcliffe Hospital Headington Oxford OX3 9DU, UK; Department of Clinical Immunology, Aalborg University Hospital, Urbansgade 32-36, DK-9000, Aalborg, Denmark; Department of Clinical Medicine, Aalborg University, Søndre Skovvej 15, Aalborg, Denmark; Department of Clinical Medicine, Aalborg University, Søndre Skovvej 15, Aalborg, Denmark; Division of Cardiovascular Medicine, Radcliffe Department of Medicine University of Oxford Level 6, West Wing John Radcliffe Hospital Headington Oxford OX3 9DU, UK; Department of Physiology, Anatomy and Genetics, University of Oxford, Sherrington Building Parks Road, OX1 3PT, Oxford, UK; Department of Physiology, Anatomy and Genetics, University of Oxford, Sherrington Building Parks Road, OX1 3PT, Oxford, UK; Division of Cardiovascular Medicine, Radcliffe Department of Medicine University of Oxford Level 6, West Wing John Radcliffe Hospital Headington Oxford OX3 9DU, UK; Division of Cardiovascular Medicine, Radcliffe Department of Medicine University of Oxford Level 6, West Wing John Radcliffe Hospital Headington Oxford OX3 9DU, UK; Division of Cardiovascular Medicine, Radcliffe Department of Medicine University of Oxford Level 6, West Wing John Radcliffe Hospital Headington Oxford OX3 9DU, UK; Division of Cardiovascular Medicine, Radcliffe Department of Medicine University of Oxford Level 6, West Wing John Radcliffe Hospital Headington Oxford OX3 9DU, UK; Sir William Dunn School of Pathology, University of Oxford, South Parks Road, Oxford, OX1 3RE, UK; Sir William Dunn School of Pathology, University of Oxford, South Parks Road, Oxford, OX1 3RE, UK; Sir William Dunn School of Pathology, University of Oxford, South Parks Road, Oxford, OX1 3RE, UK; CeMM Research Center for Molecular Medicine of the Austrian Academy of Sciences, Lazarettgasse 14, AKH BT 25.3, Vienna, Austria; Sir William Dunn School of Pathology, University of Oxford, South Parks Road, Oxford, OX1 3RE, UK; Department of Biological and Medical Sciences, Oxford Brookes University, Headington Campus Oxford OX3 0BP, UK; Institute of Clinical Sciences, School of Biomedical Sciences, College of Medical and Dental Sciences, University of Birmingham, Edgbaston, Birmingham, B15 2TT, UK; Division of Cardiovascular Medicine, Radcliffe Department of Medicine University of Oxford Level 6, West Wing John Radcliffe Hospital Headington Oxford OX3 9DU, UK; The OxAMI Study is detailed in the Supplementary Acknowledgments; Acute Vascular Imaging Centre, Radcliffe Department of Medicine, University of Oxford, John Radcliffe Hospital, Headington, Oxford, OX3 9DU, UK; CeMM Research Center for Molecular Medicine of the Austrian Academy of Sciences, Lazarettgasse 14, AKH BT 25.3, Vienna, Austria; Institute of Artificial Intelligence, Center for Medical Statistics, Informatics, and Intelligent Systems, Medical University of Vienna, Spitalgasse 23, BT88 1090, Vienna, Austria; Department of Biological and Medical Sciences, Oxford Brookes University, Headington Campus Oxford OX3 0BP, UK; Division of Cardiovascular Medicine, Radcliffe Department of Medicine University of Oxford Level 6, West Wing John Radcliffe Hospital Headington Oxford OX3 9DU, UK; The OxAMI Study is detailed in the Supplementary Acknowledgments; Acute Vascular Imaging Centre, Radcliffe Department of Medicine, University of Oxford, John Radcliffe Hospital, Headington, Oxford, OX3 9DU, UK; Department of Physiology, Anatomy and Genetics, University of Oxford, Sherrington Building Parks Road, OX1 3PT, Oxford, UK; Kennedy Institute of Rheumatology, University of Oxford, Roosevelt Dr, Headington, Oxford OX3 7FY, UK; NYU Cardiovascular Research Center, Department of Medicine, Division of Cardiology, School of Medicine, New York University School of Medicine, 435 E 30th St. New York, NY 10016, USA; Department of Pharmacology, University of Oxford, Mansfield Road, Oxford, OX1 3QT, UK; Division of Cardiovascular Medicine, Radcliffe Department of Medicine University of Oxford Level 6, West Wing John Radcliffe Hospital Headington Oxford OX3 9DU, UK; The OxAMI Study is detailed in the Supplementary Acknowledgments; Acute Vascular Imaging Centre, Radcliffe Department of Medicine, University of Oxford, John Radcliffe Hospital, Headington, Oxford, OX3 9DU, UK

**Keywords:** Exosome, Spleen, Myocardial infarction, Programming

## Abstract

**Aims:**

Acute myocardial infarction rapidly increases blood neutrophils (<2 h). Release from bone marrow, in response to chemokine elevation, has been considered their source, but chemokine levels peak up to 24 h after injury, and *after* neutrophil elevation. This suggests that additional non-chemokine-dependent processes may be involved. Endothelial cell (EC) activation promotes the rapid (<30 min) release of extracellular vesicles (EVs), which have emerged as an important means of cell–cell signalling and are thus a potential mechanism for communicating with remote tissues.

**Methods and results:**

Here, we show that injury to the myocardium rapidly mobilizes neutrophils from the spleen to peripheral blood and induces their transcriptional activation prior to arrival at the injured tissue. Time course analysis of plasma-EV composition revealed a rapid and selective increase in EVs bearing VCAM-1. These EVs, which were also enriched for miRNA-126, accumulated preferentially in the spleen where they induced local inflammatory gene and chemokine protein expression, and mobilized splenic-neutrophils to peripheral blood. Using CRISPR/Cas9 genome editing, we generated VCAM-1-deficient EC-EVs and showed that its deletion removed the ability of EC-EVs to provoke the mobilization of neutrophils. Furthermore, inhibition of miRNA-126 *in vivo* reduced myocardial infarction size in a mouse model.

**Conclusions:**

Our findings show a novel EV-dependent mechanism for the rapid mobilization of neutrophils to peripheral blood from a splenic reserve and establish a proof of concept for functional manipulation of EV-communications through genetic alteration of parent cells.

## Graphical Abstract

**Graphical Abstract:**
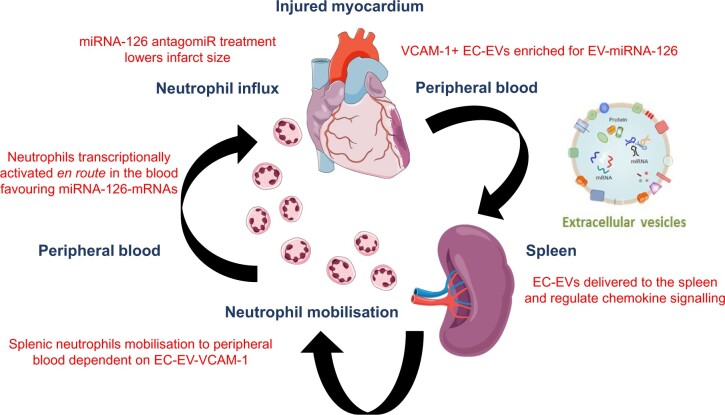

**See the editorial comment for this article ‘Extracellular vesicles selectively mobilize splenic neutrophils’, by R. Panda and P. Kubes, https://doi.org/10.1093/cvr/cvad015.**

## 1. Introduction

Acute myocardial infarction (AMI) is a substantial sterile injury that leads to a rapid increase in peripheral blood neutrophils.^1–5^ Elevated peripheral blood neutrophil number post-AMI correlates with the extent of myocardial injury, degree of cardiac dysfunction, and mortality.^1–3^ Neutrophil depletion enhances susceptibility to cardiac rupture^6^ and antibody depletion of neutrophils prior to AMI increases infarct size, enhances fibrosis, and lowers the number of M2 macrophages in the healing myocardium.^1,7^ However, inhibition of neutrophil recruitment in AMI reduces infarct size.^1^ These competing findings suggest a complex role for neutrophils in the contexts of myocardial ischaemic injury and repair.

The bone marrow is the primary site for granulopoiesis^8,9^ and has been regarded as the principal source of neutrophils that are mobilized to peripheral blood after injury.^4,10^ Mature neutrophils are held in large numbers in the haemopoietic cords, separated from the blood by the sinusoidal endothelium.^11^ In the current paradigm, these cells are retained in the marrow by the interaction of CXCR4 and CXCL12 [stromal cell-derived factor (SDF-1α)]^12^ and mobilized in response to soluble factors. Intravascular injection of a range of chemotactic factors, including leukotriene B4, C5a, interleukin-8 (IL-8),^13^ CXCL chemokines,^12,14^ and granulocyte-colony stimulating factor (G-CSF)^15,16^ can drive the rapid mobilization of neutrophils across the sinusoidal endothelium. However, numerous strands of evidence question whether chemokines derived from injured tissues are responsible for very early neutrophil mobilization *in vivo*. Intra-cardiac mRNA levels for cytokines peak 12 h after injury^17^ and pro-inflammatory proteins are very modestly elevated in coronary sinus following reperfusion therapy in AMI.^18,19^ Furthermore, *in vivo* blood chemokine profiles peak 24 h post-AMI and do not precede the rise in blood neutrophil counts in humans or mice, which occurs within 2 h in mice following injury,^1,7^ whereas a large majority of rodent AMI studies investigating neutrophils dynamics following AMI have focused on neutrophil elevations 6–24 h post-injury.^1,4^ Moreover, a putative source of chemokine generation in the acutely ischaemic myocardium prior to neutrophil infiltration has not been identified.

These observations suggest that neutrophils may be mobilized from alternative reserves following injury and by mechanisms that are not dependent on chemokines. One possible source is extramedullary haematopoiesis in the spleen^20^ from where neutrophils are mobilized to peripheral blood following bacterial infection.^21^ By analogy, it is known that monocytes are deployed from a splenic reserve following sterile injury in mice,^22^ and that this can be driven by extracellular vesicles (EVs) that are derived from the vascular endothelium.^23^

EVs are membrane-enclosed envelopes^24^ that are actively secreted by many cell types.^25–27^ These vesicles bear bioactive cargo that includes proteins and microRNAs (miRNAs), which are derived from the parent cell. EV can alter the biological function and cellular status of cells locally^28^ and remotely following liberation into the blood.^29^ Endothelial cell (EC)-derived EVs (EC-EVs) bearing vascular cell adhesion molecule-1 (VCAM-1) are elevated in the blood following AMI^23,28^ and have a role in the mobilization and transcriptional programming of splenic monocytes in AMI.^23^

Here, we sought to establish whether EC-EVs contribute to the very early mobilization and programming of neutrophils and, if so, through which of their component parts. We hypothesized that EC-EVs released during AMI would localize to neutrophils in remote reserves in a process mediated by VCAM-1, which has been shown to bind to neutrophils via surface integrins.^30^ Furthermore, we reasoned that once localized to neutrophils in reserve pools, EC-EV-miRNA cargo could induce functionally relevant transcriptional programmes in those target tissues and cells prior to recruitment to the injured myocardium. An understanding of these mechanisms would immediately suggest possibilities for cell-selective immuno-modulatory interventions that are relevant in AMI and, potentially, other pathologies with an inflammatory component.

## 2. Methods

Translational studies using whole blood, plasma, plasma neutrophils, plasma EV and CMR imaging in human patients, mouse models of AMI with and without antagmiR treatment, RNA-sequencing, flow cytometry, *in vivo* EV injections, and *in vitro* studies using human and mouse ECs were employed here. Full experimental details are provided in the Supplementary material online.

### 2.1 AMI patients

All clinical investigations were conducted in accordance with the Declaration of Helsinki. The Oxfordshire Research Ethics Committee (references 08/H0603/41 and 11/SC/0397) approved human clinical cohort protocols and conformed to the principles outlined in the Declaration of Helsinki. All patients provided informed written consent for inclusion in the study.

### 2.2 LAD ligation model

All animal procedures were approved by an ethical review committee at the University of Oxford or NYU Lagone Health. Animal experiments conform to the guidelines from Directive 2010/63/EU of the European Parliament on the protection of animals used for scientific purposes or the current NIH guidelines. UK experimental interventions were carried out by UK Home Office personal licence holders under the authority of a Home Office project licence. AMI was induced in adult wild-type (WT) female C57B6/J mice as previously described.^1^ Due to the higher incidence of acute ventricular rupture in male mice.^3^ Mice were anesthetized with 4% isofluorane and maintained under 2.5% isoflurane under assisted external ventilation through the insertion of an endotracheal tube (∼200 strokes min − 1; stroke volume ∼200 μL min − 1). Buprenorphine (buprenorphine hydrochloride; Vetergesic) was delivered as a 0.015 mg/mL solution via intraperitoneal injection at 20 min before the procedure to provide analgesia. Post-AMI animals were euthanized by cervical dislocation and peripheral blood cells, splenocytes, bone marrow, and cardiac cells were isolated.

### 2.3 Statistical analysis

All values are group mean ± standard deviation (SD). Paired and unpaired two-tailed Student’s *t*-test was used to compare two groups, a one-way or two-way analysis of variance (ANOVA) or mixed model effects with *post-hoc* Bonferroni or Tukey correction was used to compare multiple group (>2) means with one, two or more independent variables. *P*-values <0.05 were considered significant. Hierarchical clustering analysis and generation of heatmap plots was performed using the pheatmap R package v1.0.12.

## 3. Results

### 3.1 Plasma neutrophil number correlates with the extent of AMI

**Figure 1:**
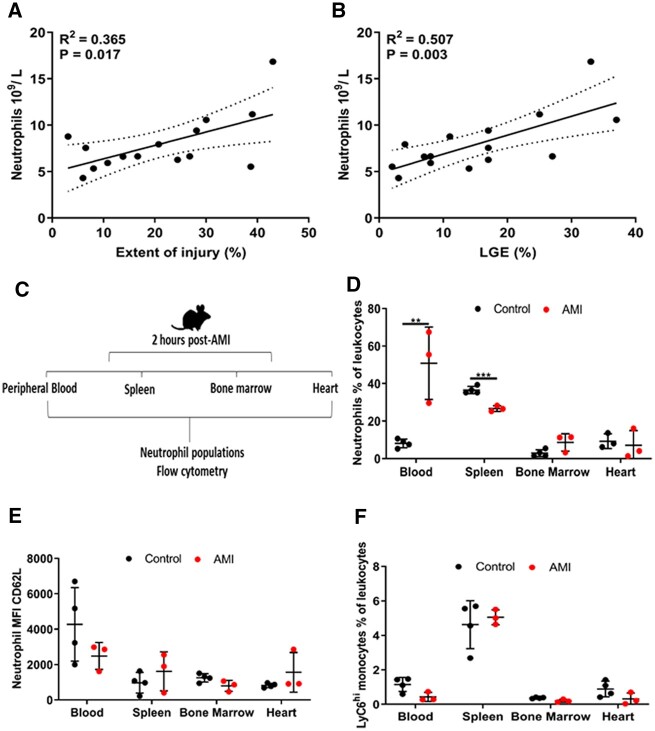
Human peripheral blood neutrophils correlate with the extent of myocardial injury in AMI. (*A*) Pearson’s correlation of peripheral blood neutrophil number (10^9^/L) in patients experiencing AMI significantly correlated with the extent of myocardial injury (T2-weight MRI) and (*B*) LGE MRI 6-months post-AMI (*n*=15). (*C*) Schematic representation of mouse AMI and tissue harvesting for flow cytometry. (*D*) Percentage of neutrophils in peripheral blood, spleen, bone marrow, and heart 2 h after AMI in mice relative to the levels of intact controls (controls *n* = 4, AMI *n* = 3). (*E*) Mean fluorescence intensity of CD62L/L-selectin on mouse neutrophils in peripheral blood, spleen, bone marrow, and heart 2 h after AMI relative to the levels of intact controls (controls *n* = 4, AMI *n* = 3). (*F*) Percentage of monocytes in peripheral blood, spleen, bone marrow, and heart 2 h after AMI in mice relative to the levels of intact uninjured controls (controls *n* = 4, AMI *n*=3). Pearson’s correlation was used in (*A*) and (*B*), dotted lines represent 95% confidence interval and an unpaired *t*-test was used in (*D*)–(*F*) for statistical analysis. Error bars represent mean ± SD **P < 0.01, ***P < 0.001.

In acute ST-segment-elevation AMI (STEMI) peripheral blood neutrophil number at the time of presentation [median time from onset of symptoms to percutaneous coronary intervention (PCI) 3 h] correlated with the extent of ischaemic injury, as determined by oedema estimation on T2-weighted magnetic resonance imaging (MRI) images obtained within 48 h of AMI (*R*^2^ = 0.365, *P* = 0.017) (*Figure 1A*) and with final infarct size, determined by late gadolinium enhancement (LGE) MRI 6 months post-AMI (*R*^2^ = 0.507, *P* = 0.003) (*Figure 1B*).

### 3.2 AMI mobilizes neutrophils from the spleen without alterations in systemic chemokines

This rapid increase in peripheral blood neutrophils is consistent with mobilization from an existing reserve. To determine the source of neutrophil mobilization in the very early hours post-AMI, we performed left anterior descending artery ligation in a mouse model of AMI and analysed cell populations from blood, spleen, bone marrow, and the heart 2 h after AMI, by flow cytometry (*Figure 1C*). AMI induced a 6.3-fold (*P* < 0.01) increase in peripheral blood neutrophils (Live, CD45^+^, CD11b^+^, Ly6G^+^) (Supplementary material online, *Figure S1*) and simultaneously lowered splenic-neutrophil number by 0.7-fold (*P* < 0.001) (*Figure 1D*). As described previously, to obtain an indication of the mobilization between reserves, we calculated a neutrophil mobilization ratio^23^ [peripheral blood neutrophils/splenic [or bone marrow] neutrophils] and found an increase in splenic-neutrophil mobilization (8.5-fold) (*P* < 0.01), but no alteration in bone marrow neutrophil number relative to control animals. There was no significant alteration in CD62L/L-selectin (which is shed during neutrophil activation) in mobilized peripheral blood neutrophils (*Figure 1E*). At this very early time point (2 h post-AMI), we found no differences in LyC6^high^ monocyte number in the peripheral blood or spleen, indicating that neutrophils mobilize from the spleen prior to splenic-monocyte mobilization^22^ (*Figure 1F*).

The prevailing paradigm is that chemokines are rapidly released from ischaemic tissues and mobilize reserves of neutrophils to the peripheral blood following AMI. To determine this in the hyper-acute phase, when splenic-neutrophils are deployed, we undertook a quantitative protein-detection array for 25 different proteins that influence neutrophil function in plasma obtained in 2 h and 24 h post-AMI in our mouse model. We found no alterations in systemic cytokines 2 h post-AMI and only found a significant increase in CCL6 24 h post-AMI (*P* < 0.05) (Supplementary material online, *Figure S2A*).

S100A8 and S100A9 are released following AMI by activated neutrophils.^4^ We measured S100A8/S100A9 heterodimer in the plasma of mice following AMI and found a significant 6.6-fold induction 2 h post-AMI, which was maintained 24 h post-AMI (6.8-fold) when compared to control mice (both, *P* < 0.01) (Supplementary material online, *Figure S1B*). These data demonstrate a rapid increase in peripheral blood neutrophils from the splenic reserve 2 h post-AMI without significant alterations in systemic plasma cytokines.

### 3.3 Human plasma EVs correlate with the extent of peripheral blood neutrophil counts in AMI and myocardial scar 6-months post-AMI

**Figure 2:**
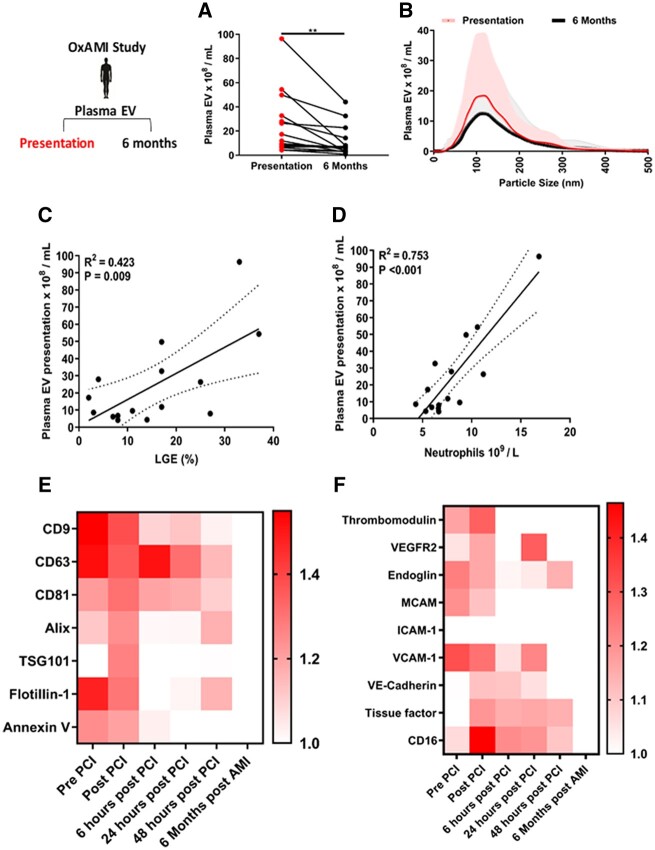
VCAM-1+ plasma EVs are elevated in peripheral blood following AMI. (*A*) Human plasma-EV number (10^8^/mL) at time of presentation following AMI and 6 months later in the same patients (*n* =15). (*B*) Size and concentration profile of human plasma EVs at time of presentation following AMI and 6 months later in the same patients (*n* =15) determined by Nanoparticle Tracking Analysis. Pearson’s correlation of human plasma EVs at time of presentation vs. and: (*C*) LGE MRI 6-months post-AMI, (*D*) number of peripheral blood neutrophils following AMI (10^9^/L) (*n* =15) in the same patients. (*E*) Heat map showing human plasma-EV markers CD9, CD63, CD81, ALIX, TSG101, flotillin-1, annexin V and (*F*) heat map showing human plasma-EV EC markers thrombomodulin, VEGFR2, endoglin, MCAM, ICAM-1, VCAM-1, VE-cadherin, tissue factor and CD16 in the same patients at: presentation, immediately following post-PCI, 6, 24, and 48 h post-PCI and 6 months post-AMI (*n* = 10 per time point). A paired *t*-test was used for statistical analysis in (*A*). Error bars in (*B*) represent mean ± SD. Heat maps in (*E*) and (*F*) are group means per time point. Values were normalized to the 6-month time point per patient. Pearson’s correlation was used in (*C*) and (*D*), dotted lines represent 95% confidence interval for statistical analysis. **P < 0.01.

In agreement with our previous findings, patients with AMI had significantly more plasma EVs at time of presentation (24.3 × 10^8^ ± 25.7 EV/mL) vs. a 6-month follow-up measurement (11.0 × 10^8^ ± 12.5 EV/mL, *P* < 0.010) but they exhibited a similar EV size distribution profile, with an elevation in EVs in the size range 100–200 nm diameter (*Figure 2A and B*). Plasma-EV number at presentation significantly correlated with the extent of myocardial scarring as determined by LGE at 6 months post-AMI (*R*^2^ = 0.423, *P* = 0.009) (*Figure 2C*). We also found a highly significant relationship between plasma-EV number and peripheral blood neutrophil number (*R*^2^ = 0.753, *P* < 0.001) at time of presentation (*Figure 2D*), consistent with a possible role for plasma EVs in neutrophil mobilization post-AMI.

### 3.4 Human VCAM-1+ plasma EVs are enriched at time of presentation with AMI

In the same patients, we determined the composition of plasma EVs at six different time points: at presentation (prior to PCI), immediately following PCI and at 6, 24, 48 h, and 6 months post-AMI using a validated high throughput immunoaffinity EV-protein array.^31^ To interpret the time course of the EV response after AMI, we normalized the EV-markers in each patient to those obtained at the 6 months post-AMI time point.

Generic EV-markers CD9, CD63, flotillin-1, CD81, ALIX, and annexin V were highly abundant in plasma EVs at the time of presentation (*Figure 2E*). At presentation plasma-EV number and EV-CD63 (*R*^2^ = 0.863, *P* = 0.001) and EV-ALIX (*R*^2^ = 0.724, *P* = 0.018) showed significant associations. Following PCI, there was augmentation of plasma-EV CD81, ALIX, TSG101, and flotillin-1, which subsided 6, 24, and 48 h post-presentation/post-PCI, approaching levels that were comparable to those at 6 months post-AMI (*Figure 2E*). Plasma EVs displayed typical morphology by transmission electron microscopy (TEM) were negative for markers of cellular contamination by histone H3 and washing of plasma EVs with phosphate-buffered saline lowered levels of apoB and albumin in isolated plasma EVs (Supplementary material online, *Figure S3A and B*).

EVs carry proteins on their surface, which can reflect their cellular origin. We determined the composition of EV in relation to EC markers, measuring thrombomodulin, vascular endothelial growth factor receptor 2 (VEGFR2), endoglin, melanoma cell adhesion molecule (MCAM), intercellular adhesion molecule-1 (ICAM-1), VCAM-1, and VE-cadherin on plasma EVs. We additionally examined tissue factor and CD16.

Plasma EVs were enriched for VCAM-1 at the time of presentation, prior to PCI and showed significant associations with plasma-EV number at presentation (*R*^2^ = 0.745, *P* = 0.013) and with EV-markers CD63 (*R*^2^ = 0.714, *P* = 0.020) and ALIX (*R*^2^ = 0.651, *P* = 0.042). VCAM-1+ plasma EV was highest at the earliest time point and diminished over time (*Figure 2F*). Plasma-EV thrombomodulin and VE-cadherin were elevated following PCI but showed no associations with plasma-EV number at presentation. Tissue factor and monocyte/neutrophil marker CD16 also showed distinct patterning post-PCI (*Figure 2F*), but with later peaks than for VCAM-1. These data suggest orchestrated rapid enrichment of plasma EVs-bearing VCAM-1 in the context of AMI.

### 3.5 Human EC-EVs are enriched for miRNA-126

**Figure 3:**
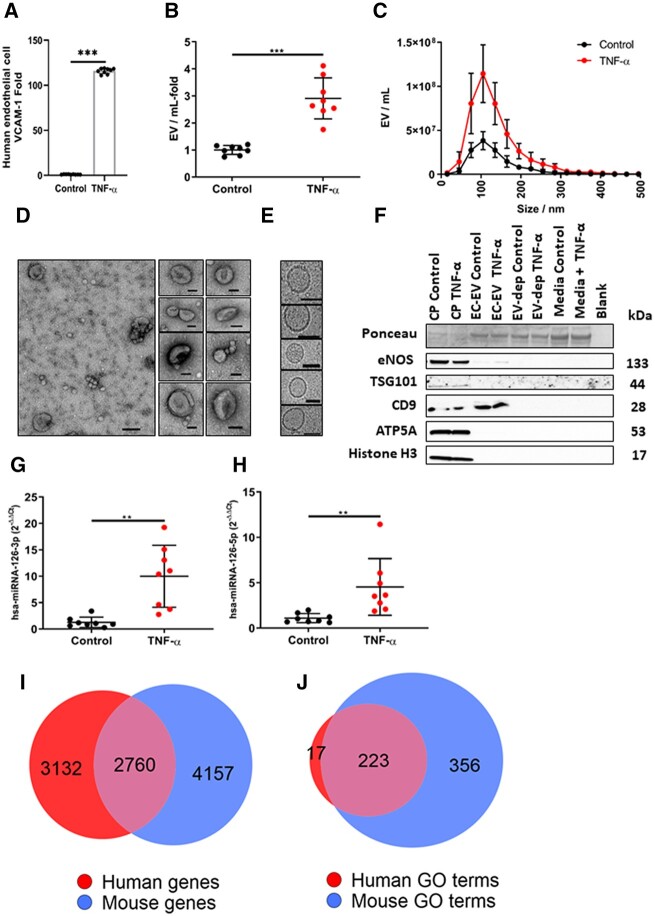
Human umbilical cord vein endothelial cells (HUVEC) release more EVs after inflammatory stimulation. (*A*) HUVECs: express more VCAM-1 following treatment with recombinant human TNF-α (10 ng/mL) (*n* = 9 per group); (*B*) release more EVs (*n* = 8 per group). (*C*) Size and concentration profile of HUVEC-derived EVs under basal conditions and after inflammatory stimulation with recombinant human TNF-α (*n* = 8 per group). (*D*) TEM of HUVEC-derived EVs (scale bar 100 nm) and (*E*) cryo-TEM HUVEC-derived EVs (scale bar 50 nm). (*F*) Ponceau stain and western blot of HUVEC-derived EV from basal and after inflammatory stimulation with TNF-α for eNOS, TSG101, CD9, ATP5A, and Histone H3. HUVEC cell pellets, EV-depleted cell culture supernatants (EV-dep), and cell culture media that was not exposed to cells (control media) were used as controls. EC-EV miRNA levels of (*G*) hsa-miRNA-126-3p and (*H*) hsa-miRNA-126-5p under basal conditions and after inflammatory stimulation with TNF-α (*n* = 8 per group). miRNA-126-mRNA targets in human and mouse and their target pathways. (*I*) Euler plot of miRNA-126-mRNA targets from TargetScanHuman, TargetScanMouse, miRWalk, miRDB for human and the mouse. (*J*) Euler plot of GO terms for miRNA-126-mRNA targets for the human and mouse. Shape areas are approximately proportional to number of genes. An unpaired *t*-test was used in (*A*), (*B*), (*C*), (*G*), and (*H*) for statistical analysis. Error bars represent mean ± SD **P < 0.01, ***P < 0.001.

In order to probe the function of EVs derived exclusively from activated ECs, we studied an established model of primary human umbilical cord vein ECs *in vitro*. Compared with basal conditions, treatment of ECs with pro-inflammatory tumour necrosis factor-α (TNF-α) activated ECs as evidenced by enhanced VCAM-1 protein expression (*P* < 0.001) (*Figure 3A*) and increased EV production (*P* < 0.001) (*Figure 3B and C*), with a significant increase in small EVs, of similar size range (100–200 nm) to those found to increase in patients with AMI. EC-EVs displayed typical EV morphology (*Figure 3D and E*), the EV-protein marker CD9 (*Figure 3F*) and were positive for endothelial nitric oxide synthase (eNOS).

EC-EVs derived from pro-inflammatory stimulations show significant enrichment for miRNA-126-3p (*P* < 0.010) (*Figure 3G*) and miRNA-126-5p (*P* < 0.010) (*Figure 3H*), consistent with previous observations of changes in miRNA, measured in the unselected plasma-EV pool, following AMI^23^ at a time point consistent with elevated VCAM-1+ plasma EVs and prior to PCI.

### 3.6 miRNA-126-mRNA targets cluster selectively in neutrophil motility pathways

To explore the potential role of EC-EV-miRNA-126, we employed *in silico* techniques, curating miRNA-126 putative-mRNA target genes from three separate miRNA–mRNA target prediction databases for human and mouse.^32–34^

We determined whether the mRNAs putatively regulated by miRNA-126 for the human, mouse or the overlap gene set (targeted in both the human and mouse) (*Figure 3I and J* and Supplementary material online, *Tables S1 and S2*) were present in Gene Ontology (GO) terms for neutrophil function. miRNA-126-putative-mRNA targets were significantly overrepresented when compared by Fisher’s exact test to neutrophil pathway GO terms for neutrophil migration (GO: GO1990266) and neutrophil chemotaxis (GO: GO0030593) in the human (both *P* < 0.001), the mouse (both *P* < 0.001), and the overlap gene set (both *P* < 0.001) (Supplementary material online, *Table S3*). Whereas, other neutrophil GO terms, such as neutrophil-mediated killing of a fungus (GO: GO0070947), neutrophil clearance (GO: GO0097350), and regulation of neutrophil-mediated cytotoxicity (GO: 0070948) were not enriched (Supplementary material online, *Table S3*), suggesting a possible role for EC-EV-miRNA-126 in orchestrating processes related to neutrophil mobilization post-AMI.

### 3.7 AMI alters human and mouse neutrophil transcriptomes

**Figure 4:**
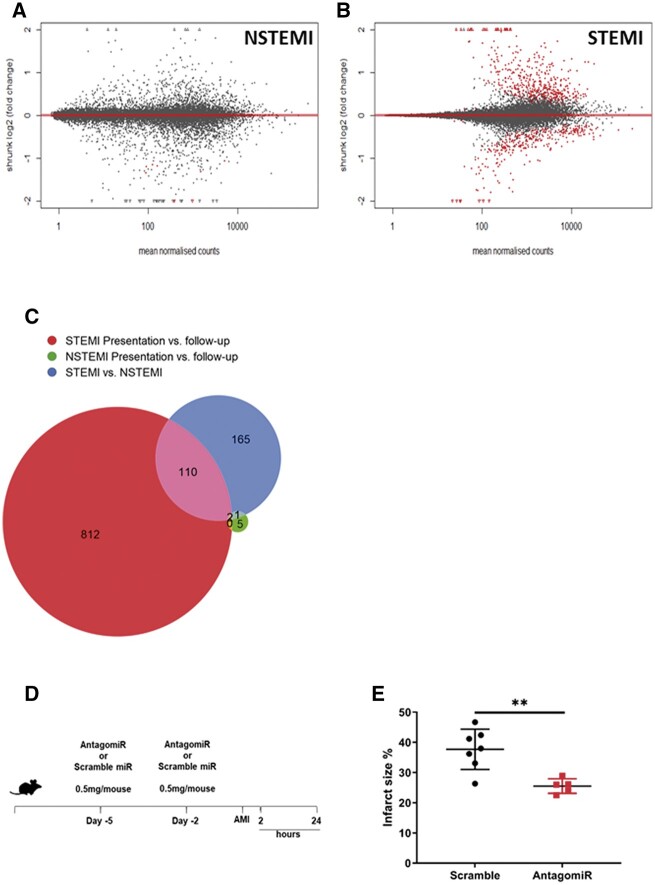
RNA sequencing of human peripheral blood neutrophils. STEMI and NSTEMI patients at the time of presentation vs. a control sample obtained from the same patients 1 month post-AMI (*n* = 3 per group). MA plots show differential transcriptome at the time of presentation vs. a control sample obtained from the same patients 1 month post-AMI in (*A*) NSTEMI and (*B*) STEMI patients. Significantly altered genes are highlighted in red. (*C*) Euler plot showing similarity and differences in the number of differentially expressed (DE) genes in NSTEMI and STEMI patients at time of presentation vs. 1 month follow-control samples or between all NSTEMI and all STEMI patients (*n* = 3 per group). (*D*) miRNA-126 antagomiR treatment of WT mice prior to induction of AMI. (*E*) TTC staining of the myocardium 24 h post-AMI in scramble and antagomiR treated mice (scramble *n* = 7 and antagomiR *n* = 5 per group). Significant DE genes in (*A*)–(*C*) were determined by adjusted *P*-values below the 5% FDR threshold. Error bars represent mean ± SD **P < 0.01.

To determine whether neutrophil transcriptomes are altered post-AMI, we obtained peripheral blood neutrophils from newly recruited patients presenting with STEMI (*N* = *3*) and non-STEMI (NSTEMI) control patients (*N* = *3*) at time of presentation and matched-control samples 1 month later. STEMI patients had a greater number of differentially expressed genes at time of presentation vs. NSTEMI control patients (STEMI 933 genes vs. NSTEMI 8 genes) (*Figure 4A–C*).

To further understand the potential target pathways for the differentially enriched genes in blood neutrophils following AMI, we used GO term enrichment analysis and Reactome pathway analysis^35^ ranked by false discovery rate (FDR)-adjusted *P*-values. GO analysis showed that differentially expressed neutrophil genes at the time of presentation favoured pathways for signal recognition particle (SRP)-dependent co-translational protein targeting to membrane (GO: 0006614 and R-HSA-1799339) (both, *P* < 0.001), co-translational protein targeting to membrane (GO: 0006613) (*P* < 0.001), and neutrophil degranulation (R-HSA-6798695) (*P* < 0.001) (Supplementary material online, *Table S4*).

Next, we used single cell (sc)-RNA-sequencing data to determine whether neutrophil populations in the peripheral blood of mice subjected to AMI exhibited similar transcriptomic alterations prior to recruitment to the heart. We found differential enrichment in neutrophil populations in the blood following AMI,^36^ which favoured pathway terms for neutrophil aggregation (GO: 0070488) (Supplementary material online, *Tables S5 and S6*) (*P* < 0.05), platelet activation (GO: 0030168) (*P* < 0.001), platelet activation, signalling, and aggregation (R-HSA-76002) (*P* < 0.001) (Supplementary material online, *Table S7*).

There were significant overlaps between the genes that are differentially expressed following AMI in the blood of the human and the mouse (*P* < 0.001) (Supplementary material online, *Table S8*) and significant similarity in target pathways (GO terms: biological process, molecular function and cellular component, and Reactome pathways) between the human and mouse (Supplementary material online, *Table S9*).

### 3.8 miRNA-126-mRNA targets are overrepresented in neutrophil transcriptomes following AMI

Human miRNA-126-mRNA targets were significantly overrepresented in human neutrophil transcriptomes at the time of injury (*P* < 0.05) (Supplementary material online, *Table S10*). Similarly, in mice, neutrophils within the myocardium (but not peripheral blood) showed differential enrichment for miRNA-126-mRNA targets (Supplementary material online, *Table S10*).

To test the functional significance of these findings, we treated WT mice with an antagomiR for miRNA-126 (*n* = 5) or a scramble control (*n* = 7) prior to induction of experimental AMI. miRNA-126 (*Figure 4D*) reduced infarct size by 12% compared with scramble control (*P* < 0.01) (*Figure 4E*) (representative images—Supplementary material online, *Figure S4A and B*).

### 3.9 EC-EVs localize to the spleen

**Figure 5:**
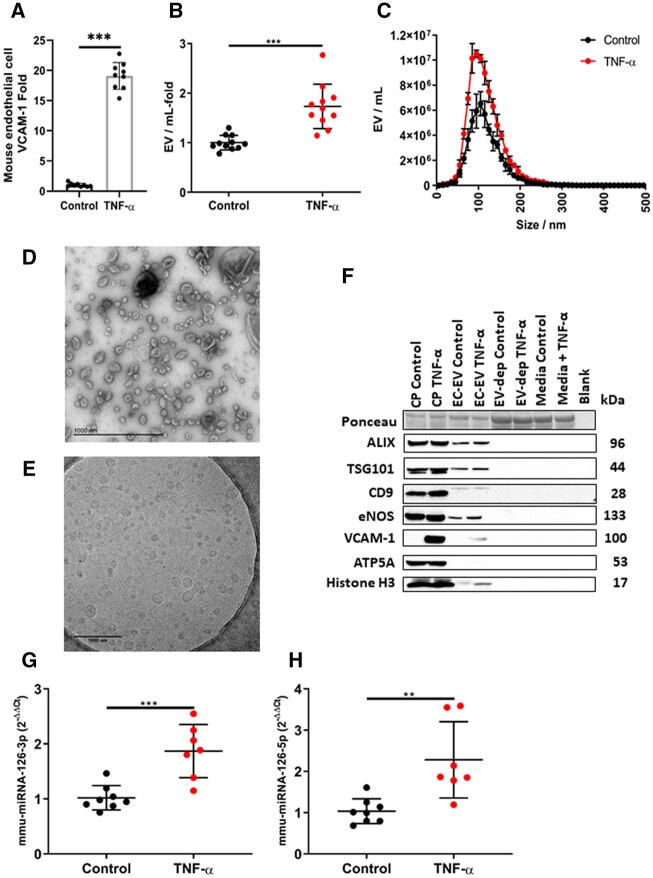
Mouse sEND.1 ECs release more EVs after inflammatory stimulation. (*A*) Mouse sEND.1 ECs express more VCAM-1 following treatment with recombinant mouse TNF-α (10 ng/mL) (*n* = 9 per group); (*B*) release more EVs (*n* = 11 per group). (*C*) Size and concentration profile of sEND.1-derived EVs under basal conditions (*n* = 3) and after inflammatory stimulation with recombinant mouse TNF-α (*n* = 4). (*D*) TEM of sEND.1-derived EVs (scale bar 1000 nm) and (*E*) cryo-TEM sEND.1-derived EVs (scale bar 1000 nm). (*F*) Ponceau stain and western blot of sEND.1-derived EV from basal and after inflammatory stimulation with TNF-α for ALIX, TSG101, CD9, eNOS, VCAM-1, ATP5A, and Histone H3. sEND1 cell pellets, EV-depleted cell culture supernatants (EV-dep), and cell culture media that was not exposed to cells (control) were used as controls. EC-EV miRNA levels of (*G*) hsa-miRNA-126-3p and (*H*) hsa-miRNA-126-5p under basal conditions (*n*= 8) and after inflammatory stimulation with TNF-α (*n* = 7). An unpaired *t*-test was used in (*A*), (*B*), (*C*), (*G*), and (*H*) for statistical analysis. Error bars represent mean ± SD **P < 0.01, ***P < 0.001.

**Figure 6:**
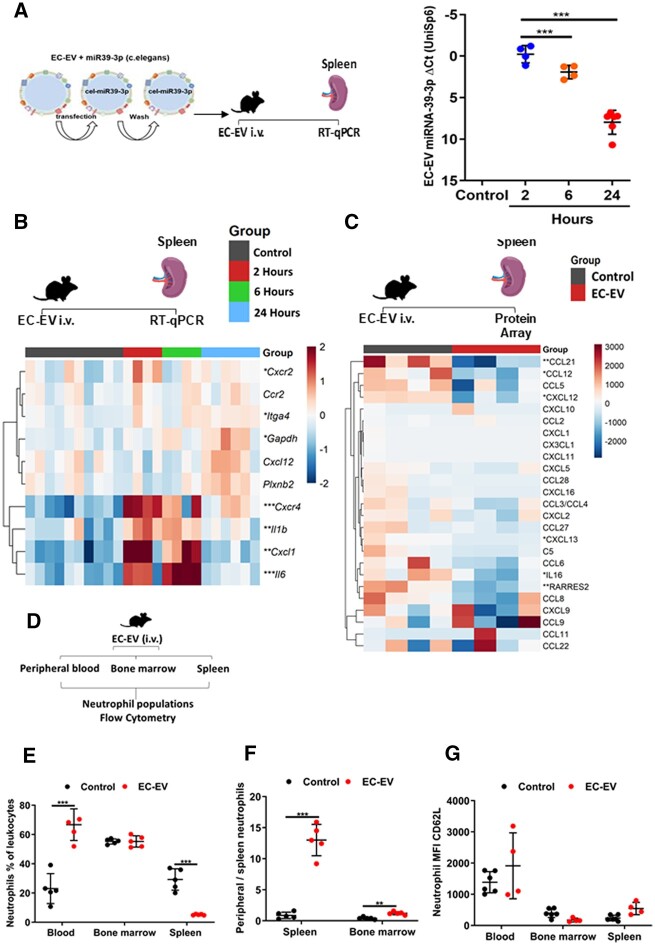
Mouse EC-EVs localize to the spleen in WT mice and influence gene and protein expression and mobilize splenic-neutrophils. (*A*) RT–qPCR detection of EC-EV labelled with miRNA-39-3p in the spleen of mice following intravenous injection of 1×10^9^ EVs by tail vein at: 2 (*n* = 4) and 6 h (*n* = 4) post-injection and control injections (*n* = 5); and 24 h (*n* = 6) post-injection and control injections (*n* = 5). Control represents a media only preparation with no EC-EVs. (*B)* Heat map showing gene expression in the spleen of mice following intravenous injection of 1×10^9^ EVs by tail vein at 2 (*n* = 4) and 6 h (*n* = 4) post-injection and control injections (*n* = 5); and 24 h (*n* = 6) post-injection and control injections (*n* = 5). Control represents a media only preparation with no EC-EVs. Data shown as ΔΔCt values normalized to row mean ΔΔCt value for each gene. (*C*) Heat map showing protein expression in the spleen of mice following intravenous injection of 1×10^9^ EVs by tail vein at 2 h (*n* = 4) post-injection. Control (*n* = 4) represents a media only preparation with no EC-EVs. Data shown are chemokine array dot blot density values normalized to mean row value for each protein. (*D*) Schematic of experiment. (*E*) Percentage of neutrophils as a proportion of the total leukocytes (live, CD45^+^, CD11b^+^, and Ly6G^+^) in peripheral blood, bone marrow, and spleen (*n* = 5 per group). (*F*) Splenic-neutrophil mobilization ratio (peripheral blood neutrophils/spleen neutrophils) shows net contributions of neutrophil reserves to mobilized peripheral blood neutrophils following intravenous injections of EC-EV (1×10^9^ EVs/mL) injections (*n* = 5 per group). (*G*) Mean fluorescent intensity of CD62L/L-selectin on neutrophils in peripheral blood, spleen, and bone marrow 2 h after (*n* = 5 per group) intravenous injections of EC-EV (1×10^9^ EVs/mL). A one-way ANOVA with *post-hoc* Bonferroni correction was used in (*A*), (*B*), and an unpaired *t*-test was used in (*C*). An unpaired *t*-test was used in (*E*)–(G). Error bars represent mean ± SD *P < 0.05, **P < 0.01, ***P < 0.001.

These accumulating data suggest that EC-EVs, enriched for miRNA-126 and VCAM-1 provide an ‘ischaemia signal’ to neutrophils in the spleen, resulting in mobilization and transcriptional activation. Accordingly, we tested whether EC-EV localized to the spleen after intravenous injection and whether there were consequent alterations in neutrophil-associated chemokine gene and protein expression. Primary mouse and human ECs release more EVs following inflammatory stimulation^23^ and hypoxia.^37^ In agreement with these data, mouse sEND.1 ECs produced EV under basal conditions and released significantly more EVs after pro-inflammatory stimulation with TNF-α (*P* < 0.001) (*Figure 5A and B*).^23^ sEND.1 TNF-α-activated ECs produced more VCAM-1 protein (*P* < 0.001) (*Figure 5C*). sEND.1-derived EVs displayed typical EV morphology (*Figure 5D and E*), EV-protein markers (ALIX, TSG101, and CD9) (*Figure 5F*) and were positive for eNOS and VCAM-1. EC-EVs derived from pro-inflammatory stimulations showed significant enrichment for miRNA-126-3p (*P* < 0.001) (*Figure 5G*) and miRNA-126-5p (*P* < 0.01) (*Figure 5H*), indicating similarities in the EC-EV response between human and mouse ECs. We labelled the mouse EC-EV by transfection with non-mammalian miRNA-39, which belongs to *Caenorhabditis elegans* and allows quantitative tracing of EVs *in vivo* (*Figure 6A*). EC-EVs accumulated preferentially in the spleen compared with bone marrow (*P* < 0.05), brain (*P* < 0.001), heart (*P* < 0.05), kidney (*P* < 0.05) (Supplementary material online, *Figure S5A*) 1 h post-injection, and remained detectable in the spleen for 2, 6 (*P* < 0.001), and 24 h (*P* < 0.001) (*Figure 6A*).

### 3.10 EC-EVs alter chemokine and protein expression in the spleen

Informed by the earlier *in silico* studies suggesting regulation of neutrophil activation and motility by miRNA-126-mRNAs, we hypothesized that EC-EV localization in the spleen would alter gene expression within spleen tissue, with a focus on CXC chemokine and cytokine activity.

EC-EVs significantly induced mRNA expression for *Cxcr2, Itag4, Gapdh* (all, *P* < 0.05), *Il-1β, Cxcl1* (both, *P* < 0.01), *Cxcr4*, and *Il-6* (both, *P* < 0.001) post-EC-EV injection (*Figure 6B*).

We further determined whether delivery of EC-EVs to the spleen altered chemokine protein levels, including for the retention chemokine CXCL12/SDF-1. In the same mice, we undertook the quantitative protein-detection array for 25 different proteins that influence neutrophil function, including CXCL12/SDF-1, CCL2,^38^ and CCL3,^39^ which are known miRNA-126-mRNA targets and CCL27/CCL28, which are predicted miRNA-126-mRNA targets. There were significant reductions in CCL21 (*P* < 0.01), CXCL13 (*P* < 0.05), chemerin/retinoic acid receptor responder protein 2 (*P* < 0.01), IL-16 (*P* < 0.05), MCP-5/CCL12 (*P* < 0.05), and CXCL12/SDF-1 (*P* < 0.05) (*Figure 6C*). These findings are consistent with a role for EC-EV-miRNA-126 in silencing genes involved in cell retention.

### 3.11 EC-EVs mobilize neutrophils from the spleen

Given the effects of the EC-EVs derived from TNF-α activated cells on gene expression and silencing of retention chemokines, we injected EC-EVs, derived from TNF-α activated ECs, intravenously into healthy WT mice vs. control media only injections with no EC-EVs (*Figure 6D*). Flow cytometry (Live, CD45^+^, CD11b^+^, Ly6G^+^) showed that EC-EVs significantly increased the number of circulating peripheral blood neutrophils (*Figure 6E*), and simultaneously lowered splenic-neutrophil numbers in the same mice (*Figure 6F*), confirming splenic-neutrophil mobilization induced by EC-EV. Consistent with our observations in AMI, we found that EC-EVs mediated greater neutrophil mobilization from the spleen (*P* < 0.001) than from the bone marrow (*Figure 6F*). As in the context of AMI, there was no alteration in CD62L/L-selectin expression in blood neutrophils (*Figure 6G*).

### 3.12 EC-EV-VCAM-1 mediates neutrophil mobilization

**Figure 7:**
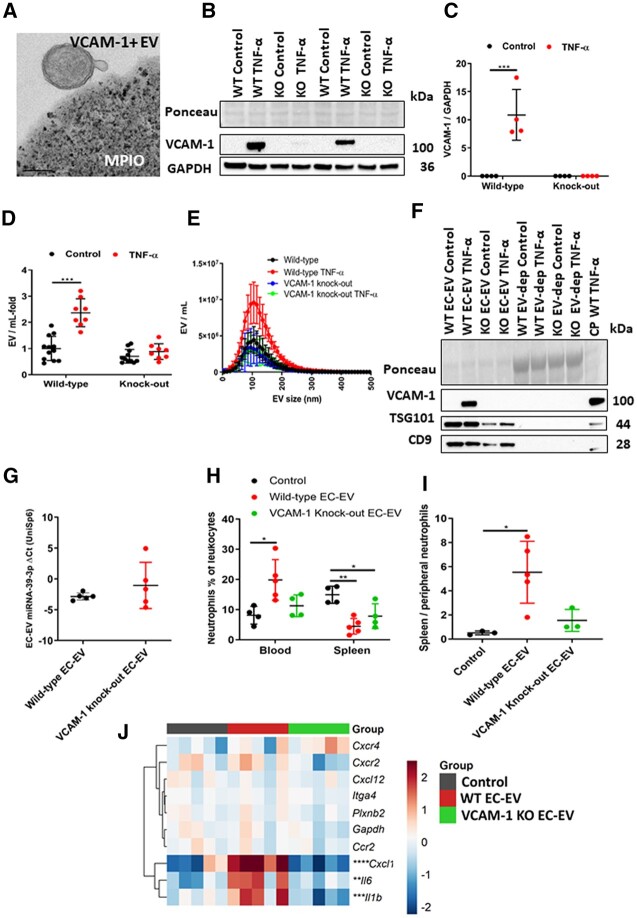
EV VCAM-1 is necessary for EC-EV splenic-neutrophil mobilization in mice. (*A*) TEM of a VCAM-1+ plasma EV bound to a magnetic bead of iron oxide conjugated with anti-human VCAM-1 antibodies, scale bar is 200 nm. (*B*/*C*) Western blot of sEND.1 WT and CRISPR-cas9 base-edited VCAM-1 KO cell pellets under basal conditions (WT *n* = 4 and VCAM-1 KO *n* = 4 per group) and after inflammatory stimulation with recombinant mouse tumour necrosis (TNF-α). (*D*) The number of mouse sEND.1 EC-EVs from WT and CRISPR-cas9 base-edited VCAM-1 KOs under basal conditions (WT *n* = 12 and VCAM-1 KO *n* = 11 per group) and after inflammatory stimulation with recombinant mouse TNF-α (*n* = 8 per group). (*E*) Size and concentration profile of mouse sEND.1 EC-EVs from WT and CRISPR/Cas9 base-edited VCAM-1 KOs under basal conditions (WT *n* = 12 and VCAM-1 KO *n* = 11 per group) and after inflammatory stimulation with recombinant mouse TNF-α (*n* = 8 per group). (*F*) Ponceau stain and western blot of WT and CRISPR-case9 base-edited VCAM-1 KO sEND.1-derived EVs from basal and after inflammatory stimulation with recombinant mouse TNF-α for TSG101, CD9, and VCAM-1. Inflammatory stimulated sEND1 cell pellets and EV-depleted cell culture supernatants were used as controls. (*G*) RT–qPCR detection of WT sEND.1 and CRISPR-cas9 base-edited VCAM-1 KOs EC-EV labelled with miRNA-39-3p in the spleen of mice following intravenous injection of 1×10^9^ EVs by tail vein at 2 h post-injection (*n* = 5 per group). (*H*/*I*) Percentage of neutrophils as a proportion of the total leukocytes (live, CD45^+^, CD11b^+^, and Ly6G^+^) in peripheral blood and spleen (control and VCAM-1 KO EC-EV *n* = 4 and WT EC-EV *n* = 5 per group). (*I*) Splenic-neutrophil mobilization ratio (peripheral blood neutrophils/spleen neutrophils) shows net contributions of splenic reserves to mobilized peripheral blood neutrophils following intravenous injections of WT or CRISPR-cas9 base-edited VCAM-1 KO EC-EVs 1×10^9^ EVs by tail vein at 2 h post-injection. Control represents a media only preparation with no EC-EVs (control and VCAM-1 KO EC-EV *n* = 3 and WT EC-EV *n* = 5 per group). (*J*) Heat map showing mRNA expression in the spleen of mice following intravenous injection of WT or CRISPR-cas9 base-edited VCAM-1 KO EC-EVs 1×10^9^ EVs by tail vein at 2 h post-injection. Control represents a media only preparation with no EC-EVs (*n* = 5 per group). Data shown as ΔΔCt values normalized to row mean ΔΔCt value for each gene. One-way (*H*–*J*) and two-way (*B*–*E*) ANOVA with *post-hoc* Bonferroni correction was used for statistical analysis. An unpaired *t*-test was used in (*F*). Error bars represent mean ± SD *P < 0.05, **P < 0.01, ***P < 0.001, ****P < 0.0001.

VCAM-1 positive EVs increases in the immediate hours after AMI (*Figure 2G*). Similarly, ECs in culture produce EVs enriched for VCAM-1 following pro-inflammatory stimulation (*Figure 5E*). Given its well-established role in mediating interactions between activated vascular endothelium and circulating leukocytes, we hypothesized that VCAM-1 on the surface of EC-EVs might perform the converse role by mediating the capture of circulating EC-EVs by static neutrophils in the spleen. We confirmed the presence of VCAM-1 on the surface of plasma EVs using immunoaffinity capture. Magnetic beads of iron oxide were conjugated to IgG control or anti-VCAM-1 antibodies and incubated with isolated human plasma EVs. Subsequent TEM shows specific capture of VCAM-1+ plasma EVs from a heterogenous pool through EV-surface expression of VCAM-1 (*Figure 7A* and Supplementary material online, *Figures S6A–E and S7A–F*).

We next used CRISPR-Cas9 base editing of ECs to produce VCAM-1 deficient EC-EVs by introducing a stop codon in the VCAM-1 sequence. To confirm CRISPR-Cas9 editing of VCAM-1 from ECs, we stimulated WT and VCAM-1 knock-out (KO) cells with TNF-α. WT mouse ECs expressed more VCAM-1 following inflammatory stimulation (*P* < 0.001) (*Figure 7B and C*), whereas VCAM-1 KO cells did not express VCAM-1, confirming successful CRISPR-Cas9 base editing in ECs. VCAM-1 KO cells released EC-EV under basal conditions similar to WT cells (*Figure 7D and E*) but VCAM-1 KO ECs did not release more EVs following inflammatory stimulation with TNF-α (*Figure 7D and E*). WT and VCAM-1 KO EC-EV were positive for EV-markers TSG101 and CD9 but only WT inflammatory-derived EC-EVs were positive for VCAM-1 (*Figure 7F*). EC-EVs derived from either TNF-α-activated WT or TNF-α-activated VCAM-1 KO cells were injected into healthy, WT mice at the same concentration (1 × 10^9^/mL EC-EVs). Using the miRNA-39-3p labelling technique (described above), we found that VCAM-1 deficient EC-EVs and WT EC-EV accumulate in the spleen at similar levels (*Figure 7G*), but VCAM-1 deficient EC-EVs did not induce alteration in gene expression that were comparable to WT EC-EVs responses in the spleen for *Il-6* (*P* < 0.001), *Il-1β* (*P* < 0.05), and *Cxcl1* (*P* < 0.05) (*Figure 7J*). Deletion of VCAM-1 in EC-EVs prevented mobilization of splenic-neutrophils to peripheral blood when compared to WT VCAM-1+ EC-EVs (*Figure 7I and J*).

## 4. Discussion

Mobilization of neutrophils occurs rapidly after AMI in mice and humans^1,3,4^ and their number in peripheral blood correlates with the extent of myocardial injury.^1–3^ The bone marrow has been regarded as the principal source for neutrophils that are mobilized to peripheral blood after injury, because (i) it is the primary site for granulopoiesis;^8–10^ (ii) it contains ample reserves of mature cells; and (iii) releases neutrophils in response to injection of exogenous chemokines.^12–16^ However, the divergent timings of neutrophil mobilization (rapid)^1,7^ and chemokine elevation (delayed) *in vivo*^17,18,40^ suggest that additional processes may be involved.

Here, we have identified a previously unknown mechanism by which ischaemic injury to the myocardium signals to mobilization of neutrophils from a splenic reserve. We show that: (i) EC-EV generated under conditions of inflammation are enriched for VCAM-1, miRNA-126-3p, and miRNA-126-5p and are elevated in peripheral blood at presentation; (ii) EC-EVs are delivered to the spleen, where they alter gene and protein expression; and (iii) induce the mobilization of splenic-neutrophils to peripheral blood. Notably, (iv) these EC-EV effects are dependent on VCAM-1. Furthermore, (v) we show that neutrophil transcriptomes are differentially regulated following AMI, prior to entry into the myocardium. (vi) Targets of miRNA-126 are significantly altered in neutrophil transcriptomes post-AMI and (vii) administration of miRNA-126 antagomir significantly reduces infarct size *in vivo*.

We utilized a systemic antagomiR strategy to determine the influence of miRNA-126 on infarct size in our rodent model. Treatment of mice by intraperitoneal injection with an antagomiR by repeated injections 5 and 2 days prior to AMI surgery may result in off target effects and does not selectively interfere with neutrophil mobilization from the spleen. However, the data reported here are consistent with a role for miRNA-126 in neutrophil activation and show lower cardiac injury following antagomiR treatment, possibly through abrogated neutrophil activation or recruitment to the injured heart. Future investigations into the role of miRNA-126 in neutrophil activation in the rodent model of AMI may benefit from more selective targeting of neutrophils through genetic approaches or the use of bioengineered EC-EV for specific immunomodulation.

Mature neutrophils are held in large numbers in the haemopoietic cords through interactions with the neutrophil receptors CXCR2 and CXCR4.^11,41,42^ Loss of CXCL12 induces an increase in peripheral blood neutrophils. Injection of chemotactic factors,^13^ CXCL chemokines,^12,14^ and G-CSF^15,16^ can drive the rapid mobilization of neutrophils across the sinusoidal endothelium through alterations in CXCR4-CXCL12.

Numerous studies have shown that neutrophil elevation in the blood and myocardium within 24 h in the rodent model of AMI but neutrophils are already elevated in patient blood by the time of arrival at the hospital. Scrutiny of the relative timings of cytokine elevation after ischaemic injury in relation to neutrophil mobilization does not support their role in this early mobilization, since both the onset and peaks in neutrophil mobilization occur prior to those for cytokine elevation.^17,18,40^ IL-8 injection mobilized neutrophils from the bone marrow,^13^ but after reperfusion in AMI, even in blood from the coronary sinus (undiluted myocardial effluent), the elevation is modest (0.1-fold).^18,19^ Furthermore, we calculate that the absolute concentration based on these physiological measurements is ∼2–3 orders of magnitude less than the concentration used to elicit neutrophil mobilization in mice.^14^ Finally, neutrophils are the first cells to arrive in the acutely injured tissue. Neutrophil depletion dampens plasma chemokines levels following AMI^7^ and in a mouse air pouch model.^43^ It is not clear which other cells in the profoundly ischaemic myocardium could be capable of the rapid synthesis of chemokines, that would be of sufficient magnitude to mediate neutrophil mobilization from a remote site, such as the bone marrow. The sympathetic nervous system is also activated following AMI and mobilizes committed myeloid lineage cells and neutrophil progenitors from the bone marrow.^44^ However, unlike terminally differentiated neutrophils, blood numbers of myeloid lineage committed cells and neutrophil progenitors do not peak until >6 h post-AMI, subsequent to the increase in peripheral blood neutrophils.

By contrast, numerous studies have shown that hypoxia promotes the rapid (<30 min) release of EVs by ECs.^37^ We show that activated ECs in culture liberate large amounts of EV that contain VCAM-1 in their membranes. Using CRISPR/Cas9 genome base editing of cultured ECs, we generated VCAM-1-deficient EV and showed that while VCAM-1 was not essential for splenic localization, its absence removed the ability of EV to provoke the rapid mobilization of neutrophils. Importantly, EVs are taken up rapidly and selectively by the spleen where they become locally concentrated,^23,45^ unlike chemokines, which have a systemic effect.

Following injury to the myocardium there is a marked increase in VCAM-1-bearing EVs. VCAM-1 is a glycoprotein, which is expressed on activated endothelium and has a well-established role in the recruitment of circulating leukocytes by binding integrins,^30,46,47^ including CD49d.^48^ Therefore, our findings suggest an efficient signalling system, in which neutrophils are activated and mobilized by engaging VCAM-1-bearing EVs that are taken up in the spleen, having been released remotely from activated endothelium. A subsequent interaction between neutrophils in circulation and static VCAM-1 on activated ECs mediates their recruitment to the original site of injury.

Deficiency of VCAM-1 by CRISPR/Cas9 in ECs impaired EC-EV release following TNF-α stimulation. The underlying mechanism for this remains unknown, but cellular integrins, such as MAC-1 form important signalling pathways for EV biogenesis in neutrophils^49^ and similarly, VCAM-1 may be necessary for inflammation induced EC-EV biogenesis.

The recruitment of neutrophils to the injured myocardium is an essential step in tissue response to injury and repair^1,7^ and thus modulating the neutrophil response raises possibilities for immuno-modulatory interventions in selected inflammatory pathologies, including AMI. Peripheral blood neutrophils are elevated at time of presentation with AMI in patients and rapidly increase in peripheral blood following AMI in mice. Here, we show that splenic-neutrophils are rapidly mobilized to peripheral blood by EC-EV-bearing VCAM-1. These findings complement the current paradigm in which neutrophils are liberated from bone marrow reserves through elevations in blood chemokines. We demonstrate a novel and efficient signalling mechanism between the injured heart microvasculature and the spleen. ECs are ideally placed for the rapid release of EVs to peripheral blood during ischaemia. EV clearance is rapid and predominately to the spleen, which contains neutrophils in the sup-capsular red pulp. Precisely how neutrophils are retained in the spleen is not known, but our findings suggest that local chemokine signalling may be important, as delivery of EC-EVs rich in miRNA-126 down-regulates retention chemokines, including the miRNA-126 target CXCL12, and induces expression of neutrophil mobilization signals, namely CXCL1.

Importantly, we show that these processes are dependent on EV-VCAM-1, an integrin ligand with a well-documented role in immune cell recruitment. Furthermore, we make a new observation that peripheral blood neutrophils are transcriptionally activated prior to recruitment to the injured myocardium, with a bias towards miRNA-126-mRNA targets. Our bioinformatics analysis revealed SRP-dependent co-translational protein targeting to membrane as the most significantly enriched pathway that was conserved between the human and the mouse blood neutrophils following AMI. SRP is necessary for transferring newly synthesized nascent proteins from the ribosome, which are destined for cellular excretion. Enrichment of SRP-dependent pathways prior to tissue recruitment may prime neutrophils for subsequent degranulation and protein secretion. At 1 day post-AMI neutrophils in the myocardium display significant enrichment for degranulation and protein secretion.^50^ A significant enrichment for the universally conserved SRP supports our conclusion that neutrophils are transcriptionally active prior to recruitment to the injured heart. Targeting SRP may open novel opportunities to target neutrophils prior to tissue recruitment to modulate their survival and function, thereby protecting injured tissues from pro-inflammatory neutrophil mediated damage.

In conclusion, we demonstrate that the injured myocardium can rapidly mobilize splenic-neutrophils through generation and release of EC-EVs that bear VCAM-1. These findings provide novel insights into how neutrophils are mobilized to peripheral blood following ischaemic injury, without the need for immediate generation and release of chemokines. EVs are decorated in surface proteins and integrins, which allows them to interact with cells and home to specific sites.^51,52^ A functionally efficient reciprocity may operate, in which VCAM-1 on EC-EVs is required for the mobilization of splenic-neutrophils, complementing the known role of static VCAM-1 in the recruitment of circulating neutrophils to activated endothelium. Neutrophils are the first cells recruited to the ischaemic myocardium and are a major source of chemokines.^7^ Thus, the well-established mobilization of neutrophils from bone marrow reserves in response to chemokines^12–16^ represent a secondary response, which is consistent with the time course of earlier reports and with our observations that there is no rapid bone marrow mobilization in the early phase.

We have shown proof of concept that genetic manipulation can alter EV properties in functionally important ways. Immunomodulation of the neutrophil and monocyte response to AMI using EV vectors may provide therapeutic opportunities in AMI.

## Supplementary material

Supplementary material is available at *Cardiovascular Research* online.

## Supplementary Material

cvac012_Supplementary_Data

## Data Availability

RNA-Sequencing data are deposited at Gene Expression Omnibus (GSE187571) and available by contacting the corresponding author. The data underlying this article are available in Gene Expression Omnibus at https://www.ncbi.nlm.nih.gov/geo/, and can be accessed with GSE187571 and upon reasonable request to the corresponding author.
