# Supplementary material for: Rapid neutrophil mobilization by VCAM-1+ endothelial cell-derived extracellular vesicles
Source: Cardiovasc Res. 2022 Feb 4;119(1):236–51. doi: 10.1093/cvr/cvac012 (PMC10022859; doi:10.1093/cvr/cvac012)
Supplement: cvac012_Supplementary_Data [file cvac012_supplementary_data.zip › 2021_12_06_Supplementary Akbar et al Neutophils.pptx]

## Slide 1
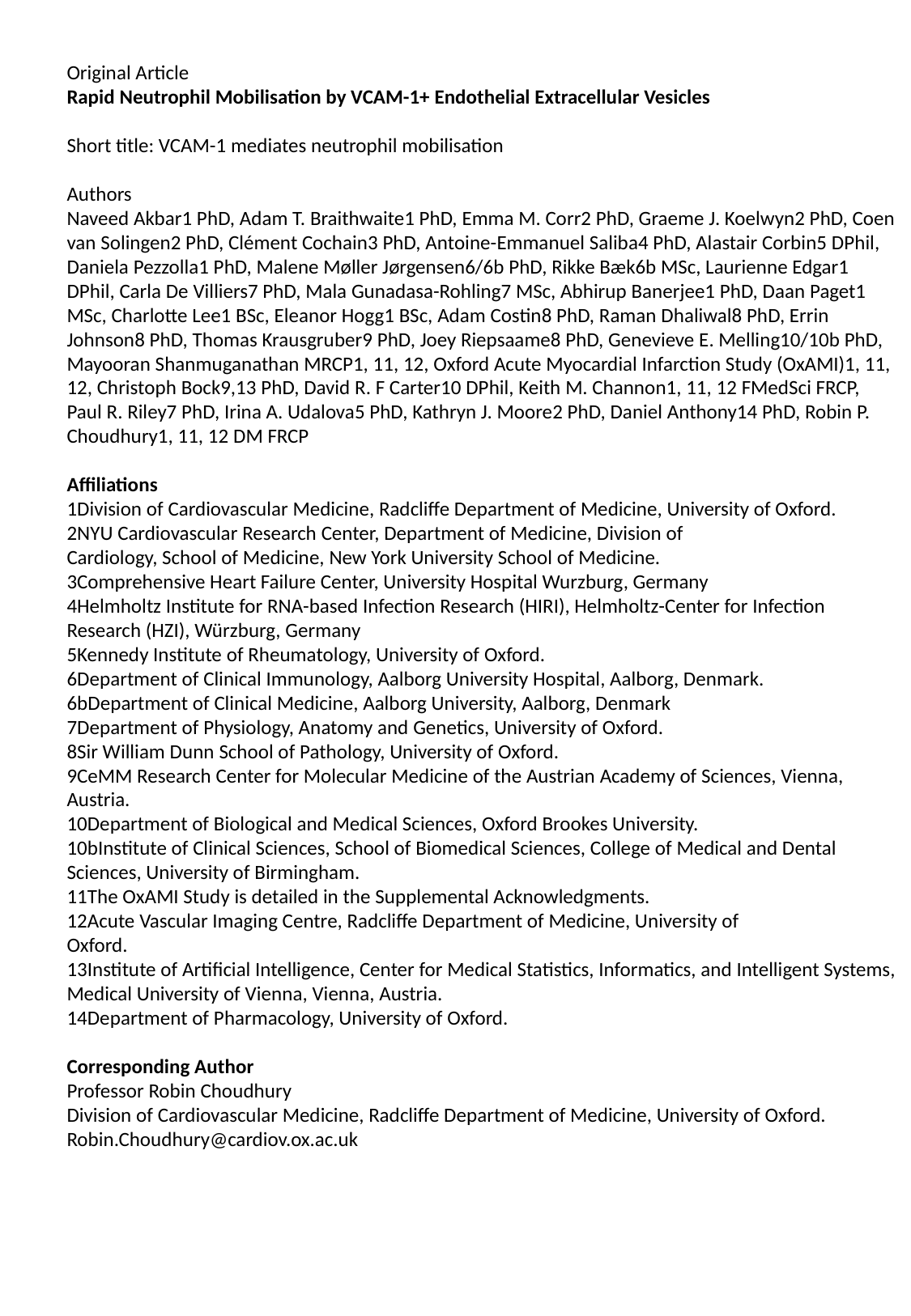

Original Article
Rapid Neutrophil Mobilisation by VCAM-1+ Endothelial Extracellular Vesicles
Short title: VCAM-1 mediates neutrophil mobilisation
Authors
Naveed Akbar1 PhD, Adam T. Braithwaite1 PhD, Emma M. Corr2 PhD, Graeme J. Koelwyn2 PhD, Coen van Solingen2 PhD, Clément Cochain3 PhD, Antoine-Emmanuel Saliba4 PhD, Alastair Corbin5 DPhil, Daniela Pezzolla1 PhD, Malene Møller Jørgensen6/6b PhD, Rikke Bæk6b MSc, Laurienne Edgar1 DPhil, Carla De Villiers7 PhD, Mala Gunadasa-Rohling7 MSc, Abhirup Banerjee1 PhD, Daan Paget1 MSc, Charlotte Lee1 BSc, Eleanor Hogg1 BSc, Adam Costin8 PhD, Raman Dhaliwal8 PhD, Errin Johnson8 PhD, Thomas Krausgruber9 PhD, Joey Riepsaame8 PhD, Genevieve E. Melling10/10b PhD, Mayooran Shanmuganathan MRCP1, 11, 12, Oxford Acute Myocardial Infarction Study (OxAMI)1, 11, 12, Christoph Bock9,13 PhD, David R. F Carter10 DPhil, Keith M. Channon1, 11, 12 FMedSci FRCP, Paul R. Riley7 PhD, Irina A. Udalova5 PhD, Kathryn J. Moore2 PhD, Daniel Anthony14 PhD, Robin P. Choudhury1, 11, 12 DM FRCP
Affiliations
1Division of Cardiovascular Medicine, Radcliffe Department of Medicine, University of Oxford.
2NYU Cardiovascular Research Center, Department of Medicine, Division of
Cardiology, School of Medicine, New York University School of Medicine.
3Comprehensive Heart Failure Center, University Hospital Wurzburg, Germany
4Helmholtz Institute for RNA-based Infection Research (HIRI), Helmholtz-Center for Infection
Research (HZI), Würzburg, Germany
5Kennedy Institute of Rheumatology, University of Oxford.
6Department of Clinical Immunology, Aalborg University Hospital, Aalborg, Denmark.
6bDepartment of Clinical Medicine, Aalborg University, Aalborg, Denmark
7Department of Physiology, Anatomy and Genetics, University of Oxford.
8Sir William Dunn School of Pathology, University of Oxford.
9CeMM Research Center for Molecular Medicine of the Austrian Academy of Sciences, Vienna, Austria.
10Department of Biological and Medical Sciences, Oxford Brookes University.
10bInstitute of Clinical Sciences, School of Biomedical Sciences, College of Medical and Dental Sciences, University of Birmingham.
11The OxAMI Study is detailed in the Supplemental Acknowledgments.
12Acute Vascular Imaging Centre, Radcliffe Department of Medicine, University of
Oxford.
13Institute of Artificial Intelligence, Center for Medical Statistics, Informatics, and Intelligent Systems, Medical University of Vienna, Vienna, Austria.
14Department of Pharmacology, University of Oxford.
Corresponding Author
Professor Robin Choudhury
Division of Cardiovascular Medicine, Radcliffe Department of Medicine, University of Oxford.
Robin.Choudhury@cardiov.ox.ac.uk

## Slide 2
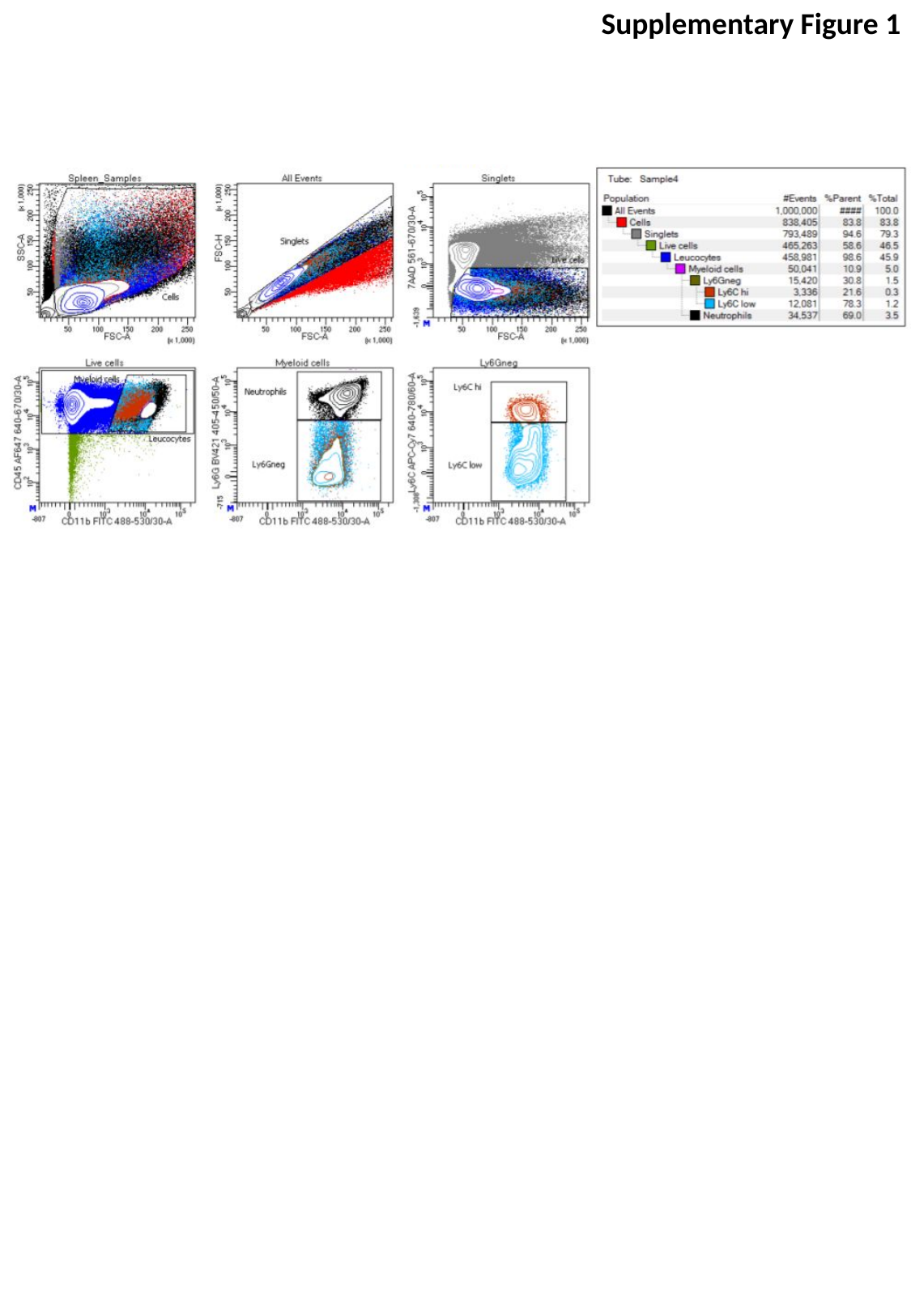

Supplementary Figure 1

## Slide 3
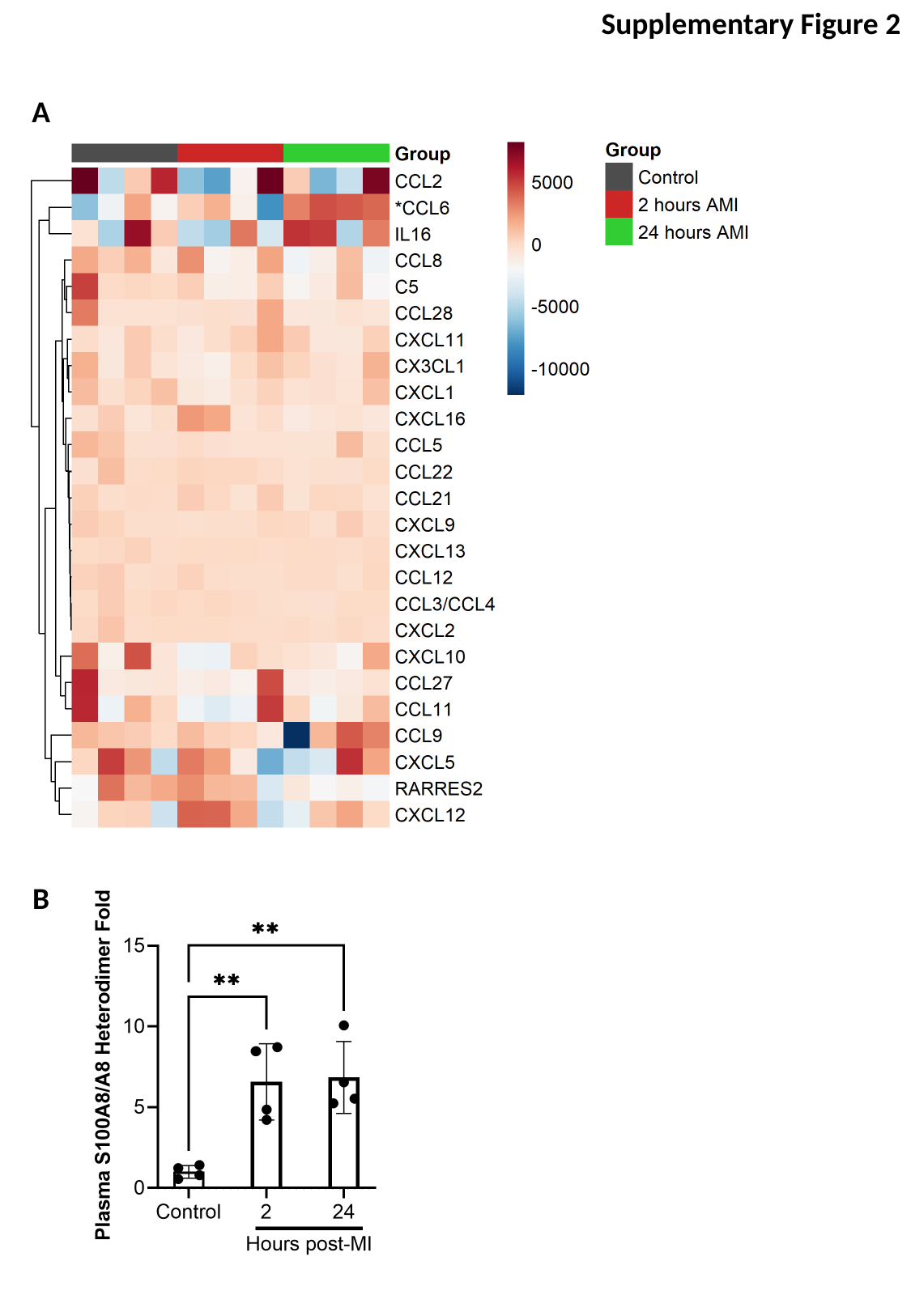

Supplementary Figure 2
A
B

## Slide 4
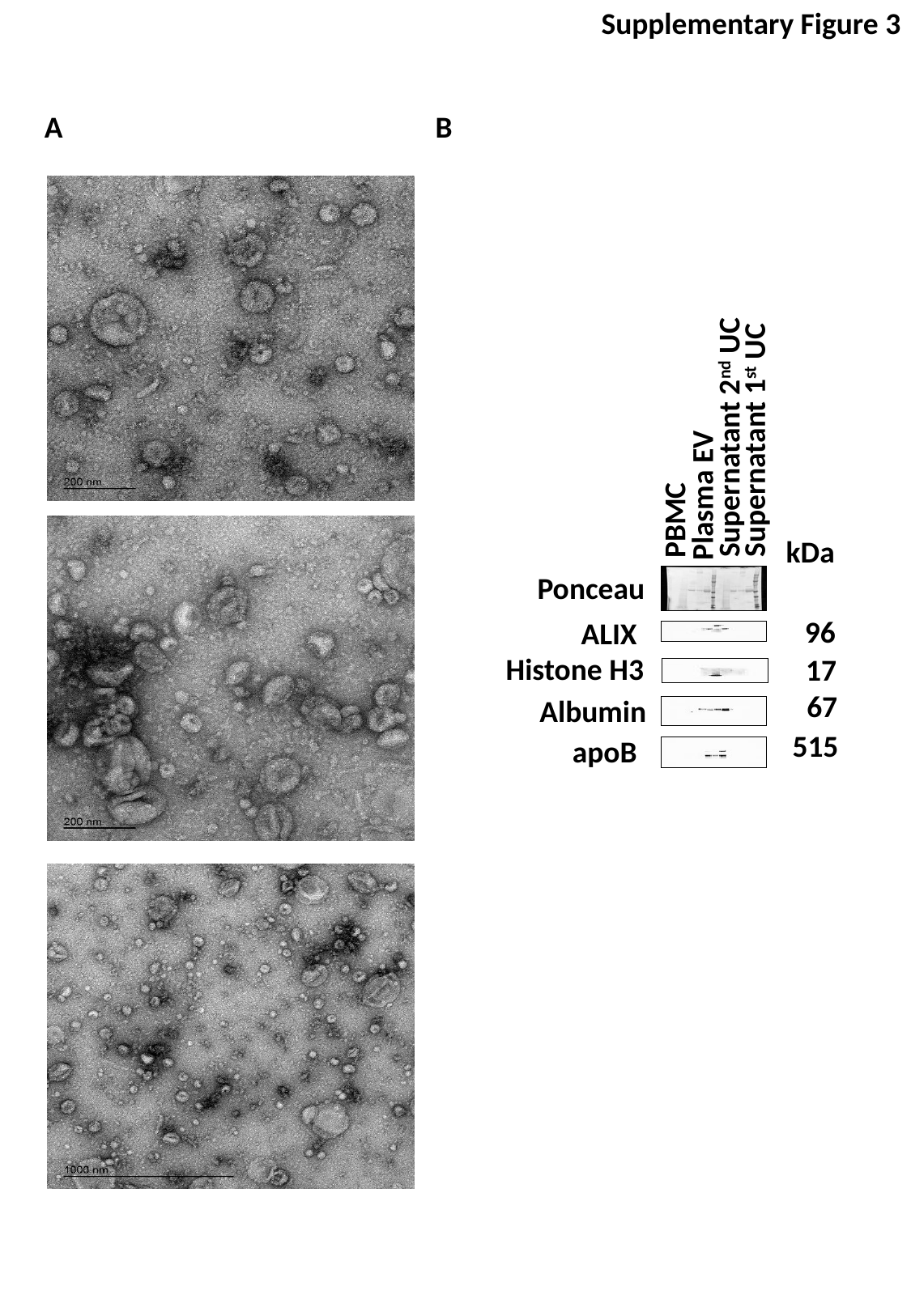

Supplementary Figure 3
A
B
Plasma EV
Supernatant 2nd UC
PBMC
Supernatant 1st UC
kDa
Ponceau
96
ALIX
Histone H3
17
67
Albumin
515
apoB

## Slide 5
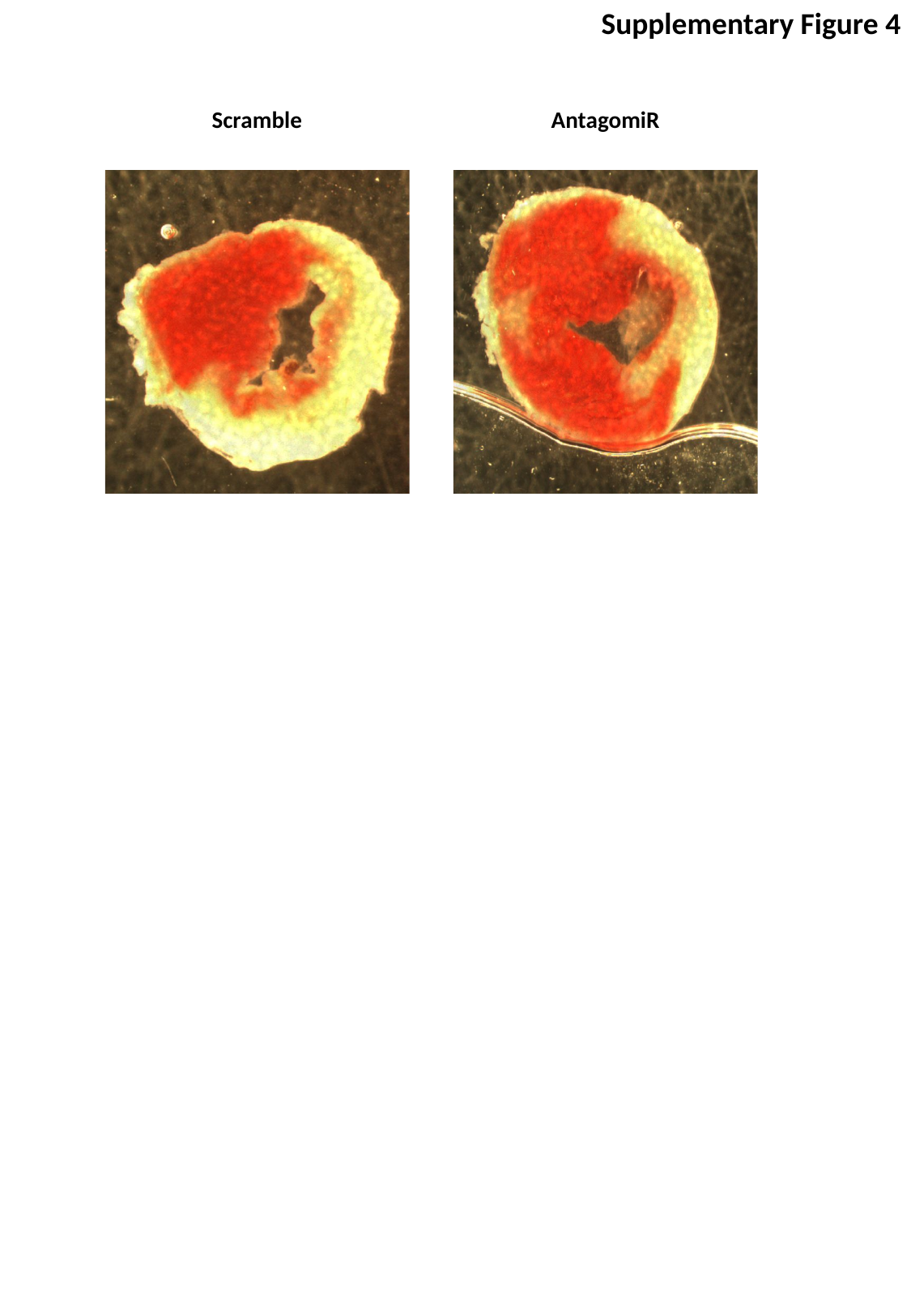

Supplementary Figure 4
Scramble
AntagomiR

## Slide 6
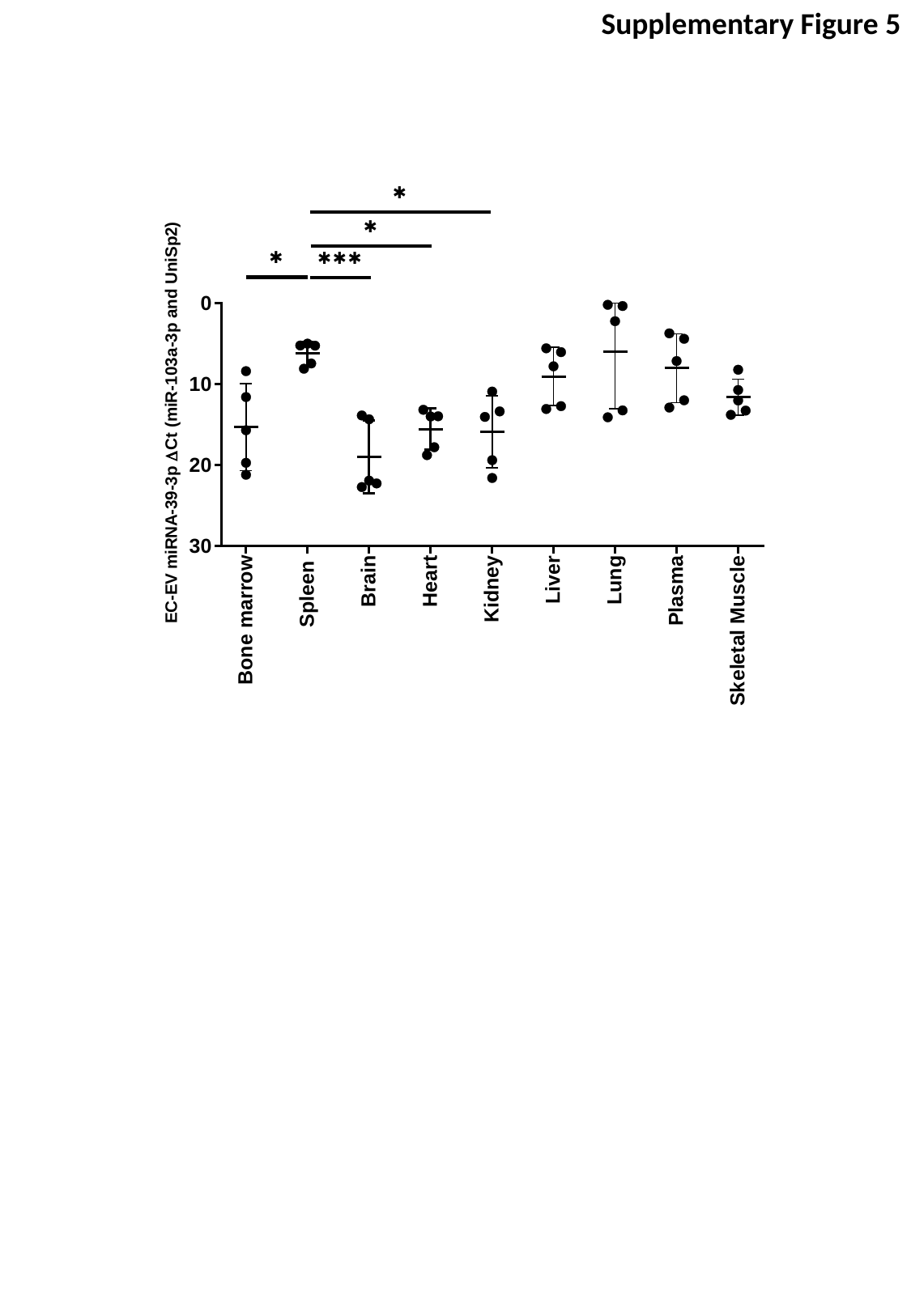

Supplementary Figure 5

## Slide 7
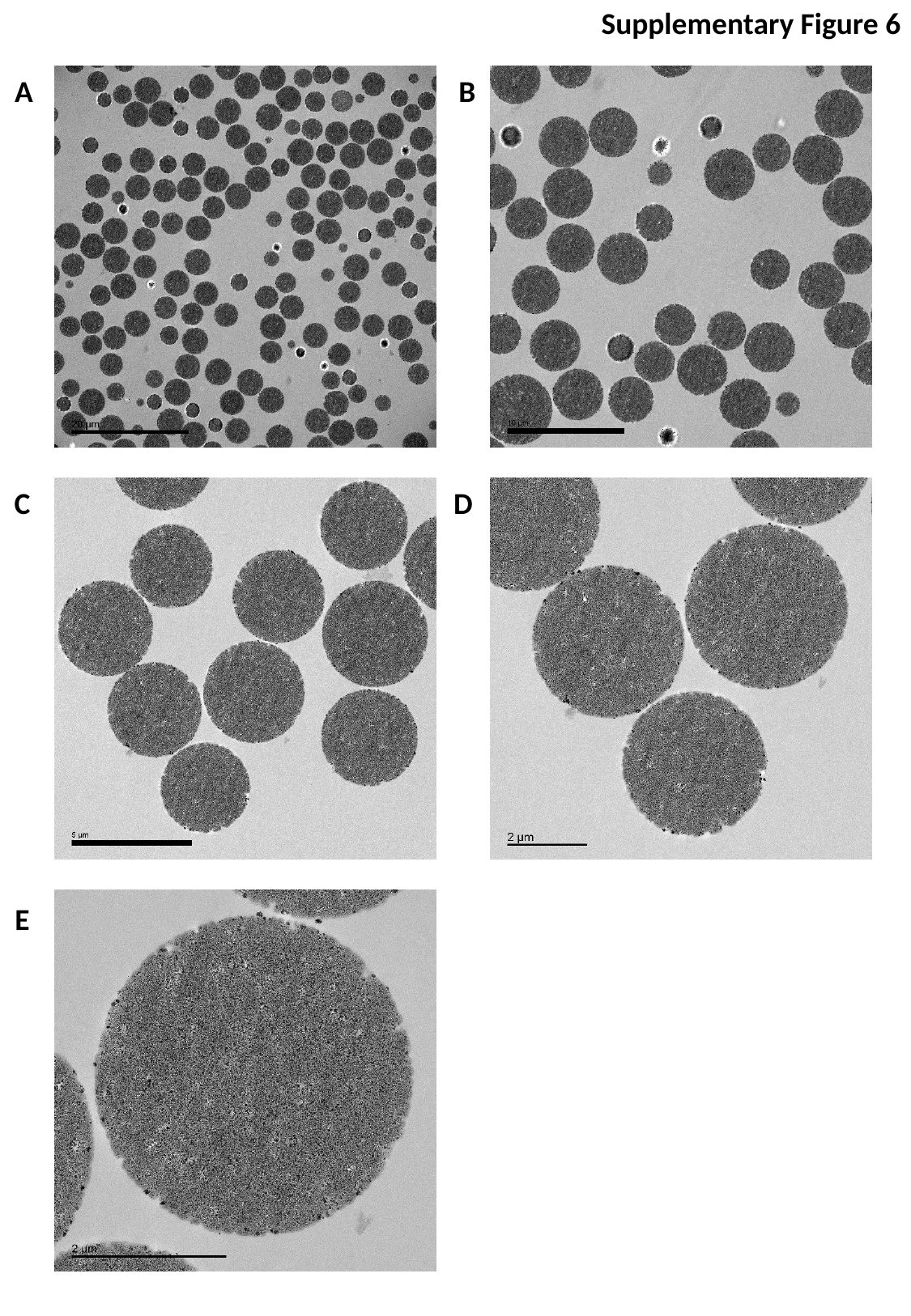

Supplementary Figure 6
A
B
C
D
E

## Slide 8
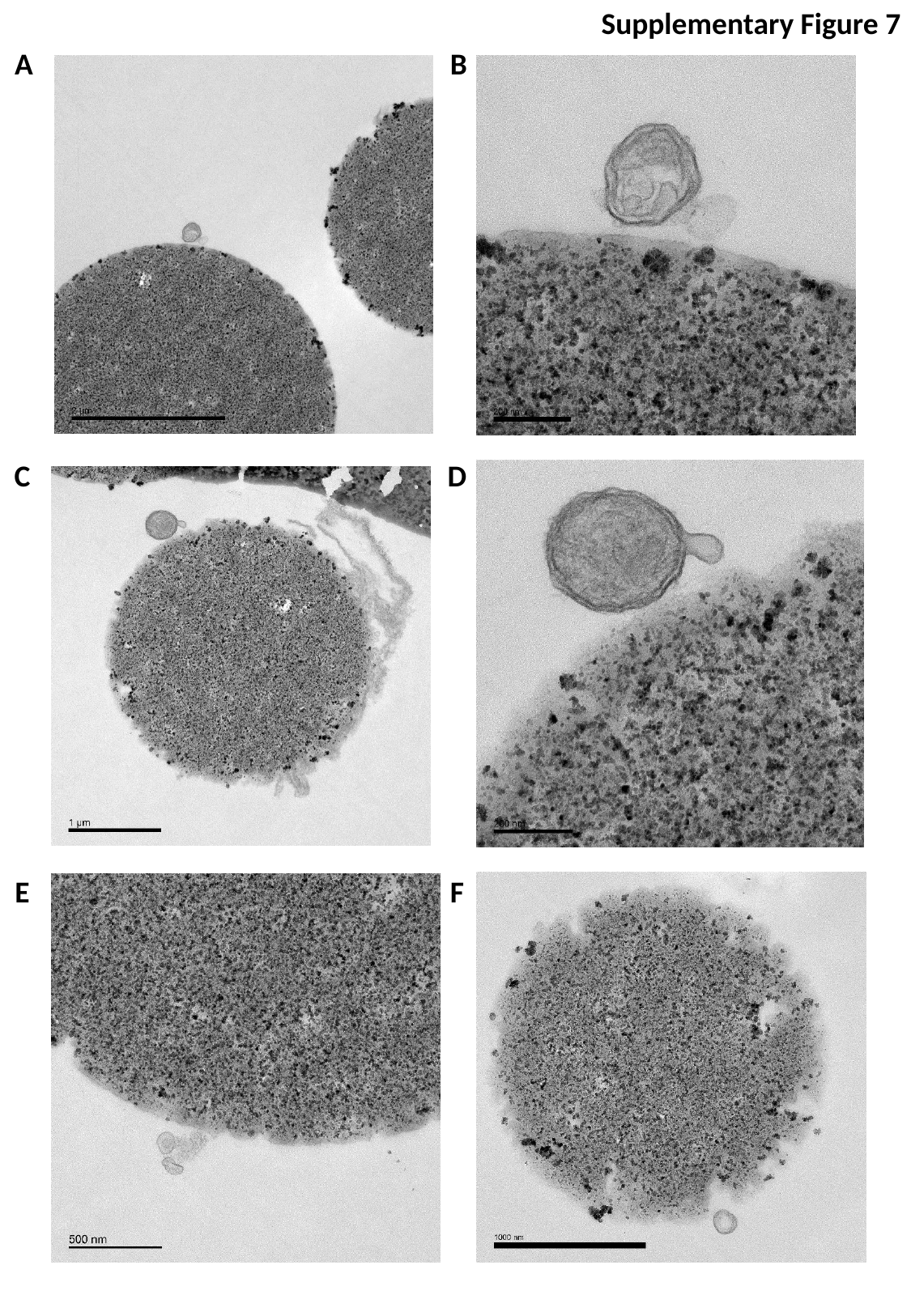

Supplementary Figure 7
A
B
C
D
E
F

## Slide 9
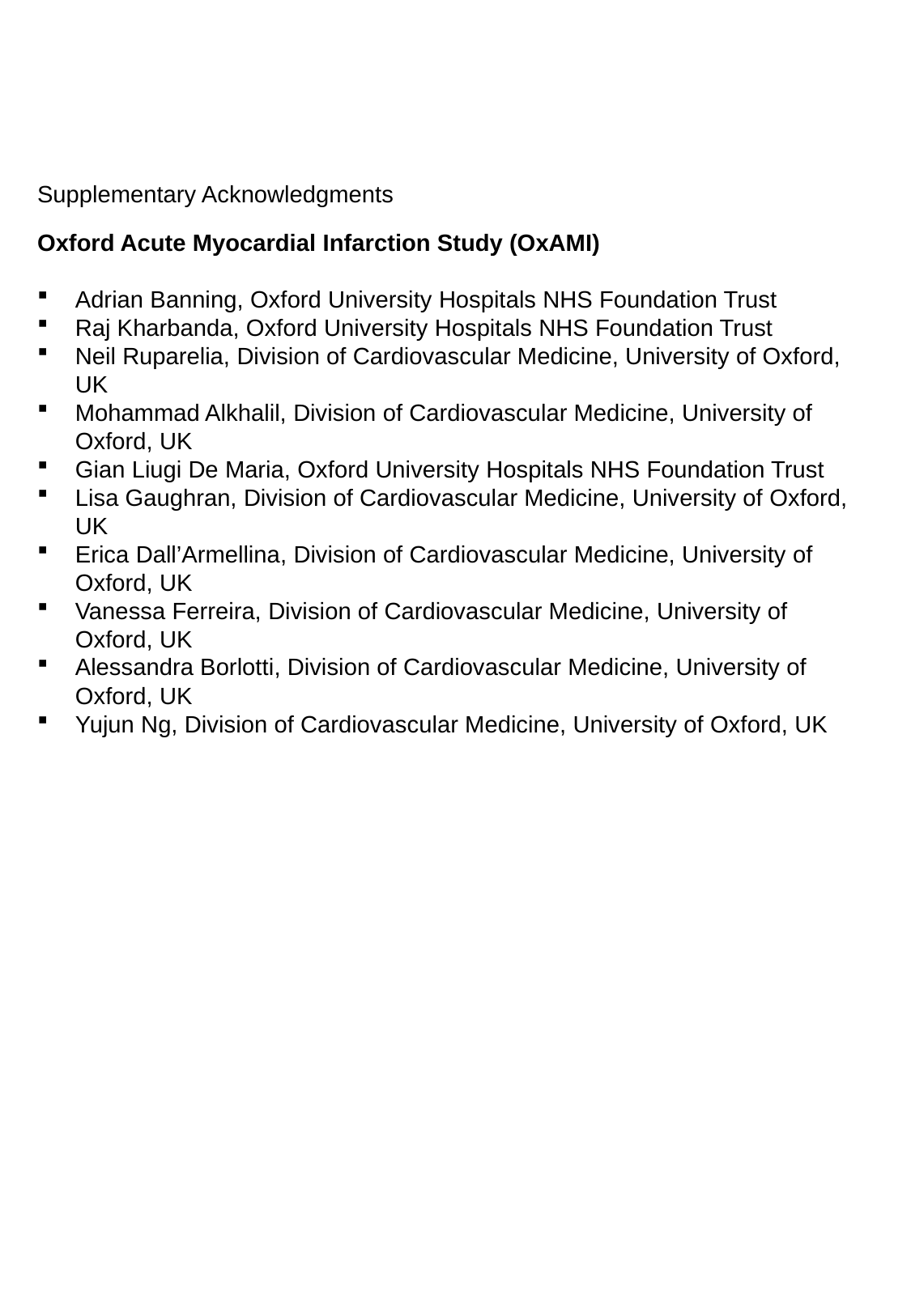

Supplementary Acknowledgments
Oxford Acute Myocardial Infarction Study (OxAMI)
Adrian Banning, Oxford University Hospitals NHS Foundation Trust
Raj Kharbanda, Oxford University Hospitals NHS Foundation Trust
Neil Ruparelia, Division of Cardiovascular Medicine, University of Oxford, UK
Mohammad Alkhalil, Division of Cardiovascular Medicine, University of Oxford, UK
Gian Liugi De Maria, Oxford University Hospitals NHS Foundation Trust
Lisa Gaughran, Division of Cardiovascular Medicine, University of Oxford, UK
Erica Dall’Armellina, Division of Cardiovascular Medicine, University of Oxford, UK
Vanessa Ferreira, Division of Cardiovascular Medicine, University of Oxford, UK
Alessandra Borlotti, Division of Cardiovascular Medicine, University of Oxford, UK
Yujun Ng, Division of Cardiovascular Medicine, University of Oxford, UK

## Slide 10
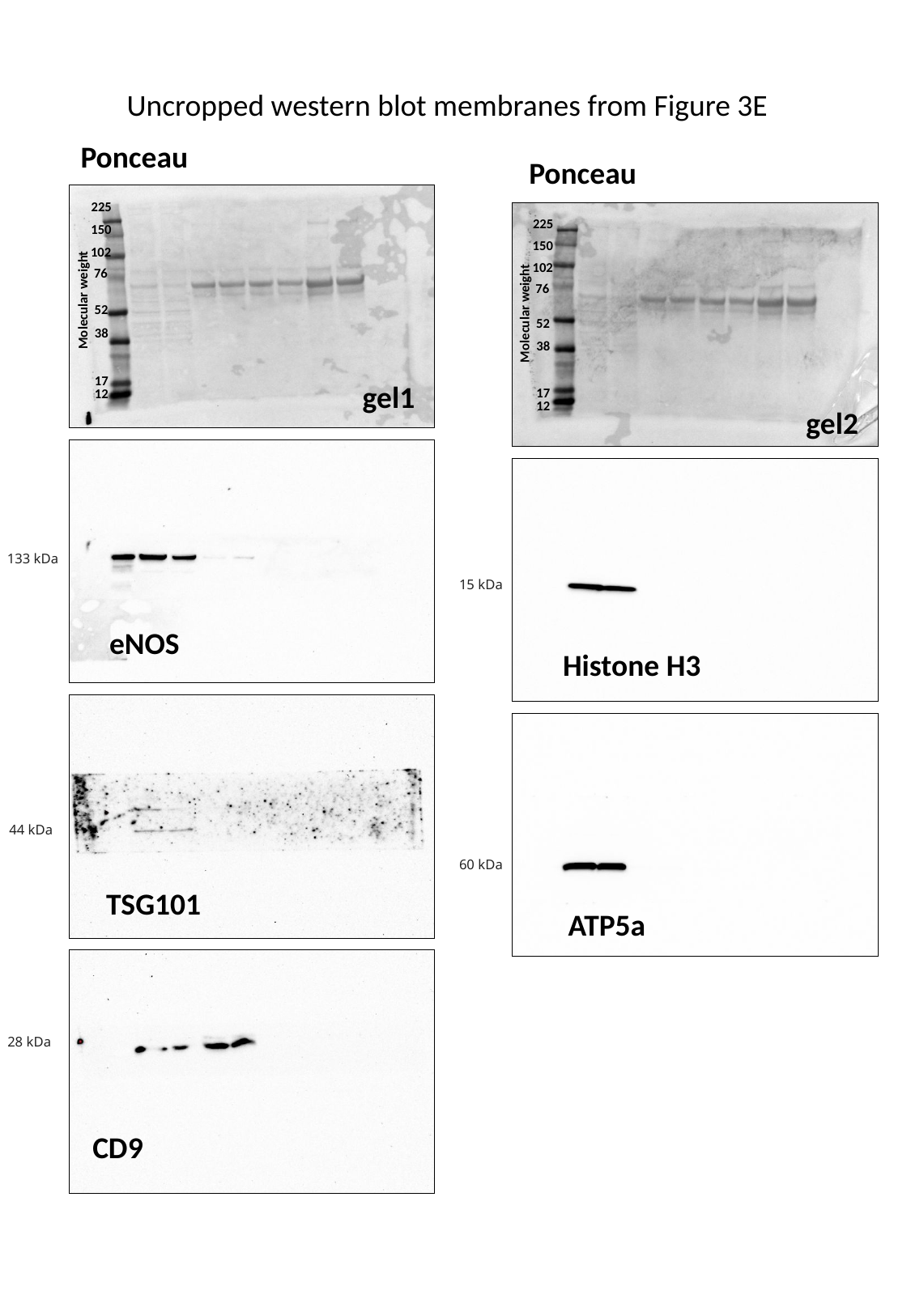

Uncropped western blot membranes from Figure 3E
Ponceau
Ponceau
gel1
eNOS
TSG101
CD9
225
150
102
76
Molecular weight
52
38
17
12
gel2
Histone H3
ATP5a
225
150
102
76
Molecular weight
52
38
17
12
133 kDa
15 kDa
44 kDa
60 kDa
28 kDa

## Slide 11
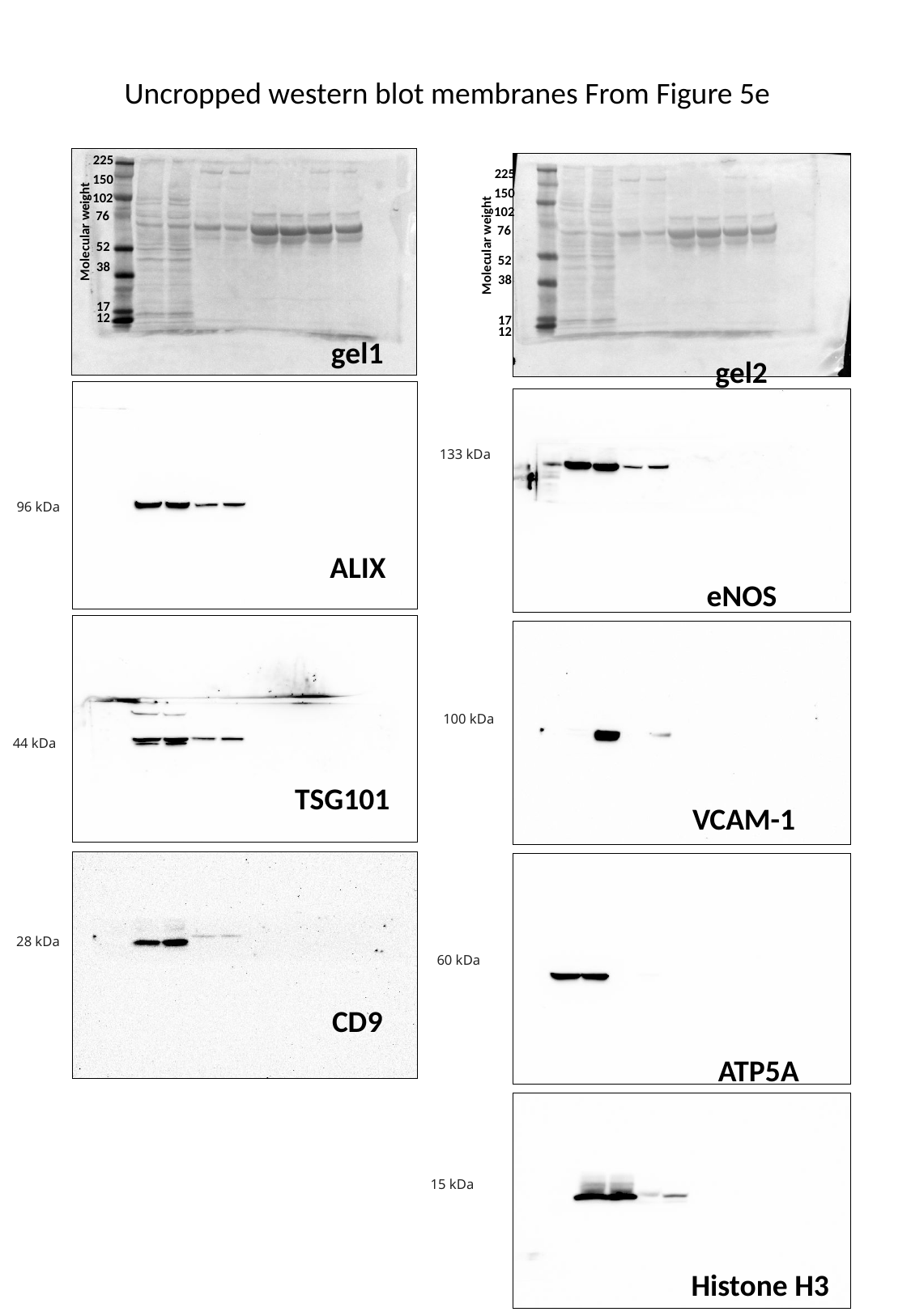

Uncropped western blot membranes From Figure 5e
225
150
102
76
Molecular weight
52
38
17
12
225
150
102
76
Molecular weight
52
38
17
12
gel1
gel2
133 kDa
96 kDa
ALIX
eNOS
100 kDa
44 kDa
TSG101
VCAM-1
28 kDa
60 kDa
CD9
ATP5A
15 kDa
Histone H3

## Slide 12
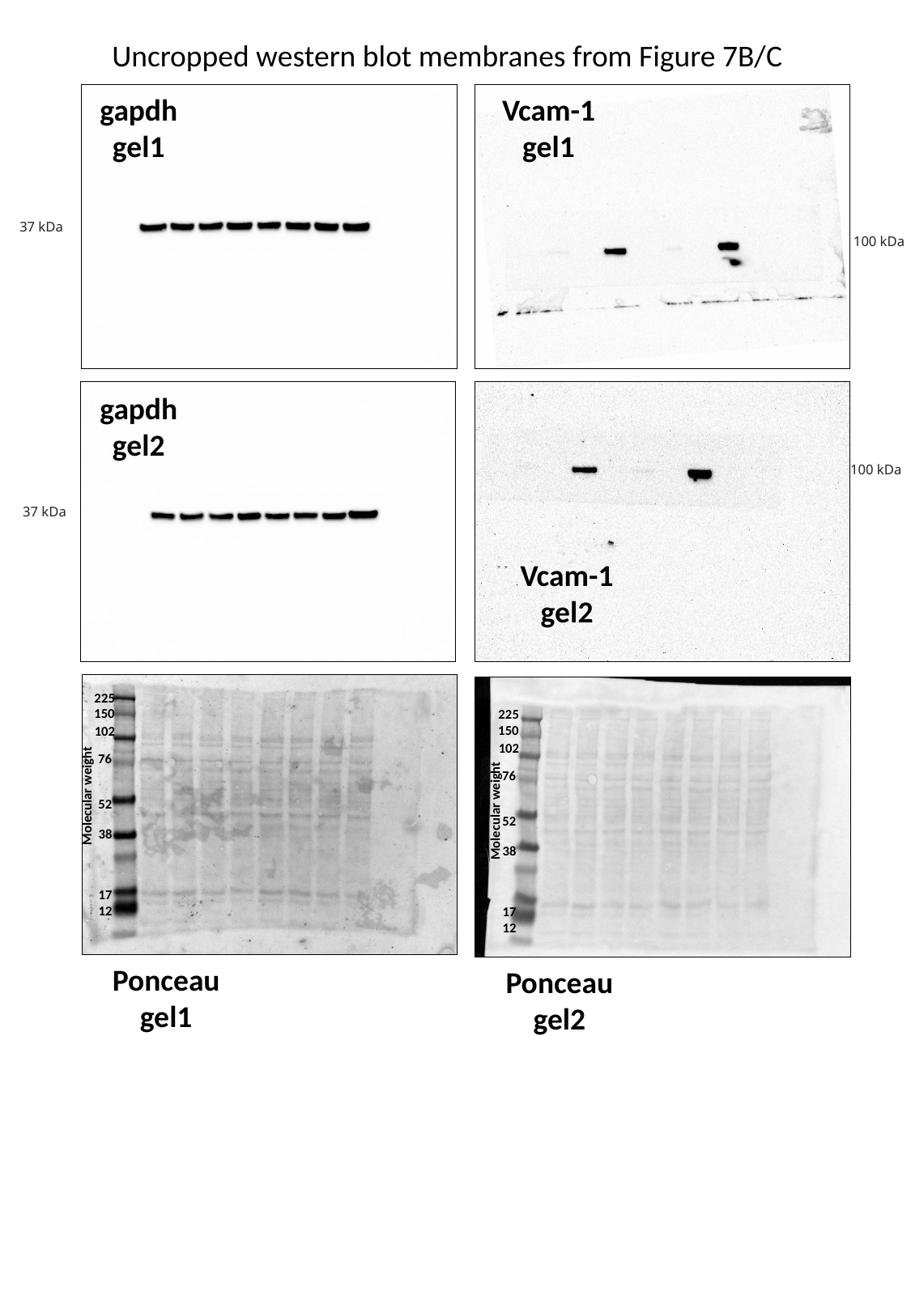

Uncropped western blot membranes from Figure 7B/C
gapdh gel1
Vcam-1
gel1
37 kDa
100 kDa
gapdh
gel2
100 kDa
37 kDa
Vcam-1
gel2
225
150
102
76
Molecular weight
52
38
17
12
225
150
102
76
Molecular weight
52
38
17
12
Ponceau gel1
Ponceau gel2

## Slide 13
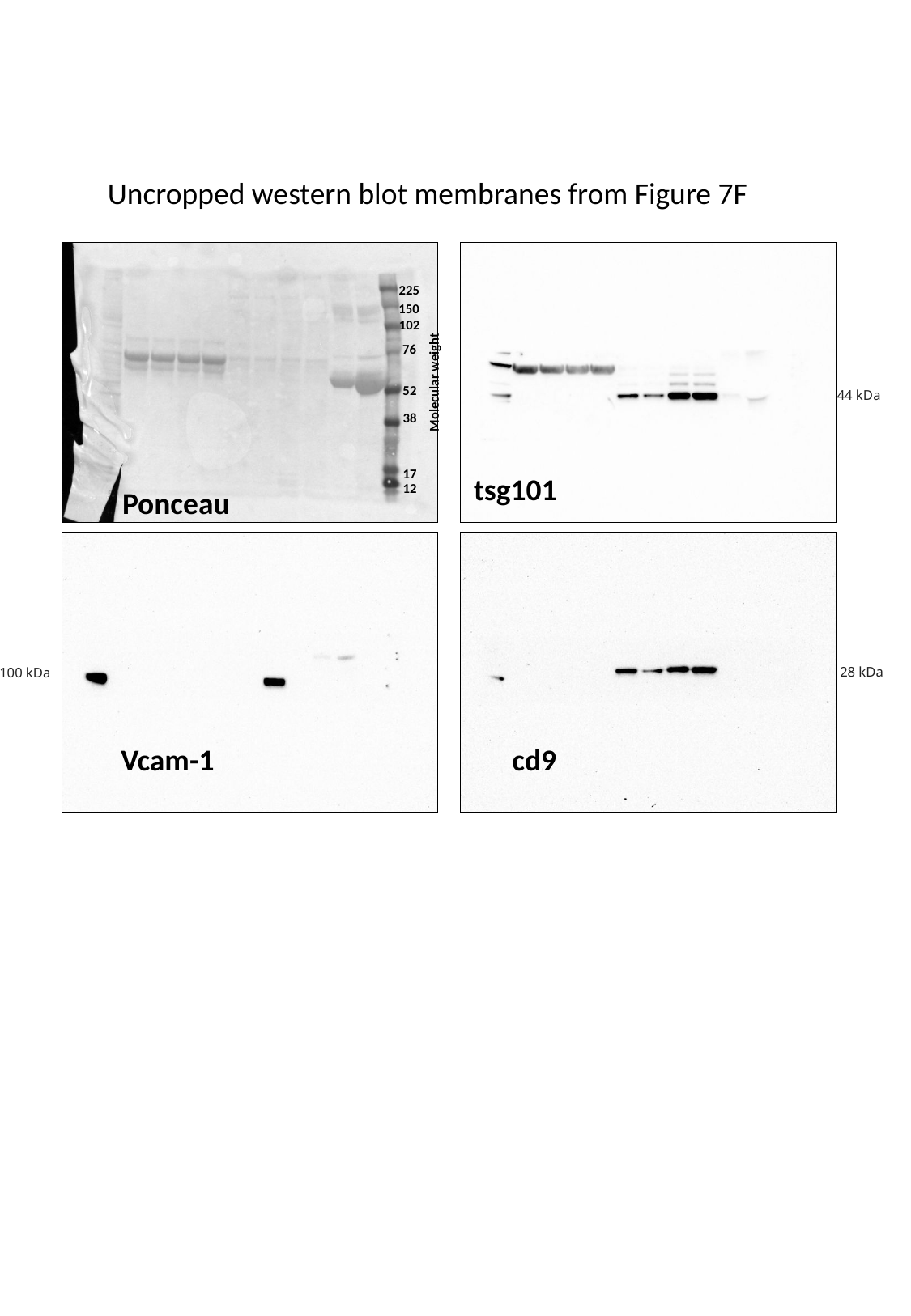

Uncropped western blot membranes from Figure 7F
225
150
102
76
Molecular weight
52
38
17
12
44 kDa
tsg101
Ponceau
28 kDa
100 kDa
cd9
Vcam-1
